# Structure and dynamics of the EGFR/HER2 heterodimer

**DOI:** 10.1038/s41421-023-00523-5

**Published:** 2023-02-13

**Authors:** Xue Bai, Pengyu Sun, Xinghao Wang, Changkun Long, Shuyun Liao, Song Dang, Shangshang Zhuang, Yongtao Du, Xinyi Zhang, Nan Li, Kangmin He, Zhe Zhang

**Affiliations:** 1 State Key Laboratory of Membrane Biology, School of Life Sciences, Peking University, Beijing, China; 2 State Key Laboratory of Molecular Developmental Biology, Institute of Genetics and Developmental Biology, Chinese Academy of Sciences, Beijing, China; 3 University of Chinese Academy of Sciences, Beijing, China; 4 Center for Life Sciences, Academy for Advanced Interdisciplinary Studies, Peking University, Beijing, China

**Keywords:** Cryoelectron microscopy, Oncogenes, Growth factor signalling

## Abstract

HER2 belongs to the human epidermal growth factor receptor tyrosine kinase family. Its overexpression or hyperactivation is a leading cause for multiple types of cancers. HER2 functions mainly through dimerization with other family members, such as EGFR. However, the molecular details for heterodimer assembly have not been completely understood. Here, we report cryo-EM structures of the EGF- and epiregulin-bound EGFR/HER2 ectodomain complexes at resolutions of 3.3 Å and 4.5 Å, respectively. Together with the functional analyses, we demonstrate that only the dimerization arm of HER2, but not that of EGFR, is essential for their heterodimer formation and signal transduction. Moreover, we analyze the differential membrane dynamics and transient interactions of endogenous EGFR and HER2 molecules in genome-edited cells using single-molecule live-cell imaging. Furthermore, we show that the interaction with HER2 could allow EGFR to resist endocytosis. Together, this work deepens our understanding of the unique structural properties and dynamics of the EGFR/HER2 complex.

## Introduction

The proteins in the human epidermal growth factor receptor (HER or ErbB) family are some of the most thoroughly studied receptor tyrosine kinases. This family consists of four members, namely EGFR (also known as HER1), HER2, HER3, and HER4. Ligand-induced homo- or hetero-dimerization of HER proteins initiates a downstream phosphorylation signaling cascade, which stimulates cell growth, proliferation, and differentiation^1^. These four HER proteins are highly associated with tumorigenesis, with EGFR and HER2 considered the most potent oncoproteins. Their overexpression is implicated in many types of cancers, including breast, lung, and gastroesophageal. Antibody- and tyrosine kinase inhibitor-based therapies targeting these two receptors have been widely used in the clinical treatment of various malignancies and are already among the most successful targeted tumor therapies to date^2,3^.

HER2 is unique within the HER family in that it does not have known ligands and cannot assemble into ligand-dependent homodimers. Thus, to facilitate downstream signaling, it must either form heterodimers with other HER proteins once their specific ligands have bound or self-assemble into ligand-independent homodimer under the condition of overexpression^1,4–6^. Among the HER2-containing heterodimers, EGFR/HER2 and HER2/HER3 are the most relevant combinations due to their impacts on cellular functions and disease^3,7–14^. The HER family homodimerization mechanisms related to extracellular ligand-binding and intracellular kinase domains have been well studied^15–22^; however, the molecular mechanisms for HER2 heterodimerization with other family members remain elusive. The cryo-EM structure of the HER2/HER3 complex was reported recently^23^, providing some initial insight into HER2-containing dimers. However, to comprehensively elucidate the structural and functional properties of distinct heterodimers, it is necessary to further investigate the EGFR/HER2 complex.

Here, we report the cryo-EM structures of the EGFR/HER2 ectodomain complex with two EGFR ligands — epidermal growth factor (EGF) and epiregulin (EREG) — at resolutions of 3.3 Å and 4.5 Å, respectively. These two structures are almost identical and present as asymmetric heterodimers. Our biochemical and cell-based experiments demonstrate that only the dimerization arm (DA) of HER2 is crucial for its heterodimerization with EGFR and subsequent initiation of downstream signals, while that of EGFR is dispensable. Using CRISPR/Cas9 genome-editing and single-molecule imaging techniques, we further explore the diffusion dynamics and interactions of endogenous EGFR and HER2 at the plasma membrane of two human breast cancer cell lines. Unlike EGFR, HER2 does not change its membrane dynamics or undergo endocytosis upon EGF activation; moreover, it helps to retain EGFR on the plasma membrane. Therefore, HER2 likely resists the rapid endocytosis and degradation of EGFR after activation, prolongs the downstream phosphorylation signals to promote cell growth and proliferation, and ultimately results in tumorigenesis. Taken together, these findings deepen our understanding of the structural and pathological properties of the EGFR/HER2 heterodimer.

## Results

### Architecture of the EGFR/HER2 ectodomain complex

**Fig. 1:**
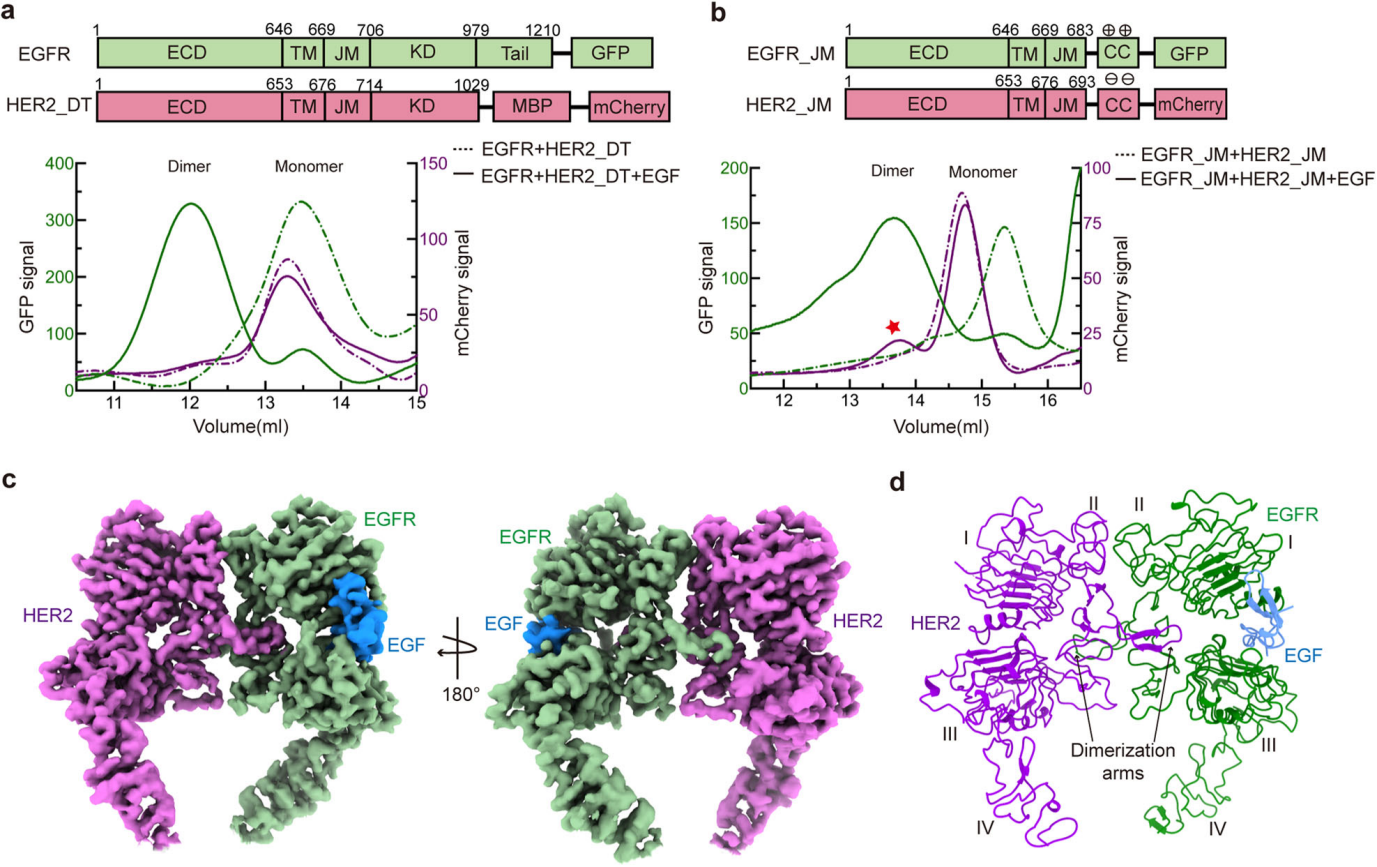
Cryo-EM structure of the EGF-bound EGFR/HER2 ectodomain complex. **a**, **b** Detection of the EGFR/HER2 dimer using FSEC. GFP- and mCherry-tags are attached to the C-termini of EGFR and HER2, respectively. HER2_DT is the tail-deleted form with a substituted MBP-tag. EGFR_JM and HER2_JM constructs only contain the ectodomain, transmembrane domain, part of the juxtamembrane domain, and the coiled-coil (CC) peptide. The basic and acidic CC peptides are attached to EGFR_JM and HER2_JM, respectively. The red pentagram in **b** indicates the peak position of EGFR_JM/HER2_JM dimer. **c** Cryo-EM map of the EGF-bound EGFR/HER2 ectodomain complex shown in two views. EGFR, EGF, and HER2 are colored in green, marine, and magenta, respectively. **d** Overall structure of the EGF-bound EGFR/HER2 heterodimer in ribbon presentation. The color code is the same as that in **c**. The four domains of EGFR and HER2 ectodomains are indicated with Roman numerals. The DAs of Domain II are also labeled.

Full-length EGFR and HER2 were coexpressed in HEK293S GnTI^–^ cells. Using fluorescence-detection size-exclusion chromatography (FSEC), we showed that EGF stimulation effectively induced the homodimerization of EGFR, but could not trigger the formation of the EGFR/HER2 complex (Fig. 1a); this result indicates that the interaction between EGFR and HER2 is much weaker than that of the EGFR homodimer, which is consistent with previous findings^24^. To stabilize the EGFR/HER2 complex, we substituted the C-terminal kinase and tail domains of EGFR and HER2 with a pair of basic and acidic coiled-coil peptides^25–27^. The FSEC results for the coiled-coil constructs showed the appearance of a HER2 dimer peak in the presence of EGF (Fig. 1b). Since HER2 does not form homodimers, this peak, therefore, represents the EGFR/HER2 heterodimer. We then purified this heterodimer using a tandem affinity purification strategy and performed the following cryo-EM study.

During 3D classification, both the EGF-bound EGFR/HER2 heterodimer and EGFR homodimer were present in our dataset, in nearly equal particle amounts. Unlike the perpendicular orientation of the EGFR homodimer, the ectodomains of the EGFR/HER2 heterodimer were tilted with respect to the detergent micelles (Supplementary Fig. S1); this structural discrepancy between the homo- and hetero-dimers helped to distinguish between these two subclasses. Finally, the complex structures of the EGF-bound EGFR/HER2 and homodimeric EGFR ectodomains were refined to 3.3 Å and 3.8 Å resolution, respectively (Fig. 1c, d; Supplementary Figs. S1–S3 and Table S1). The structure of the EGFR homodimer resembles other previously reported ones^15,28,29^ (Supplementary Fig. S3f–h), indicating that the coiled-coil peptides in our constructs would not affect the regular assembly of the receptor dimers. The relatively lower resolution of the EGFR homodimer compared to the EGFR/HER2 heterodimer might be caused by its intrinsic flexibility^29^ or the destabilization by the electrostatic repulsion between the C-terminal basic coiled-coil peptides.

**Fig. 2:**
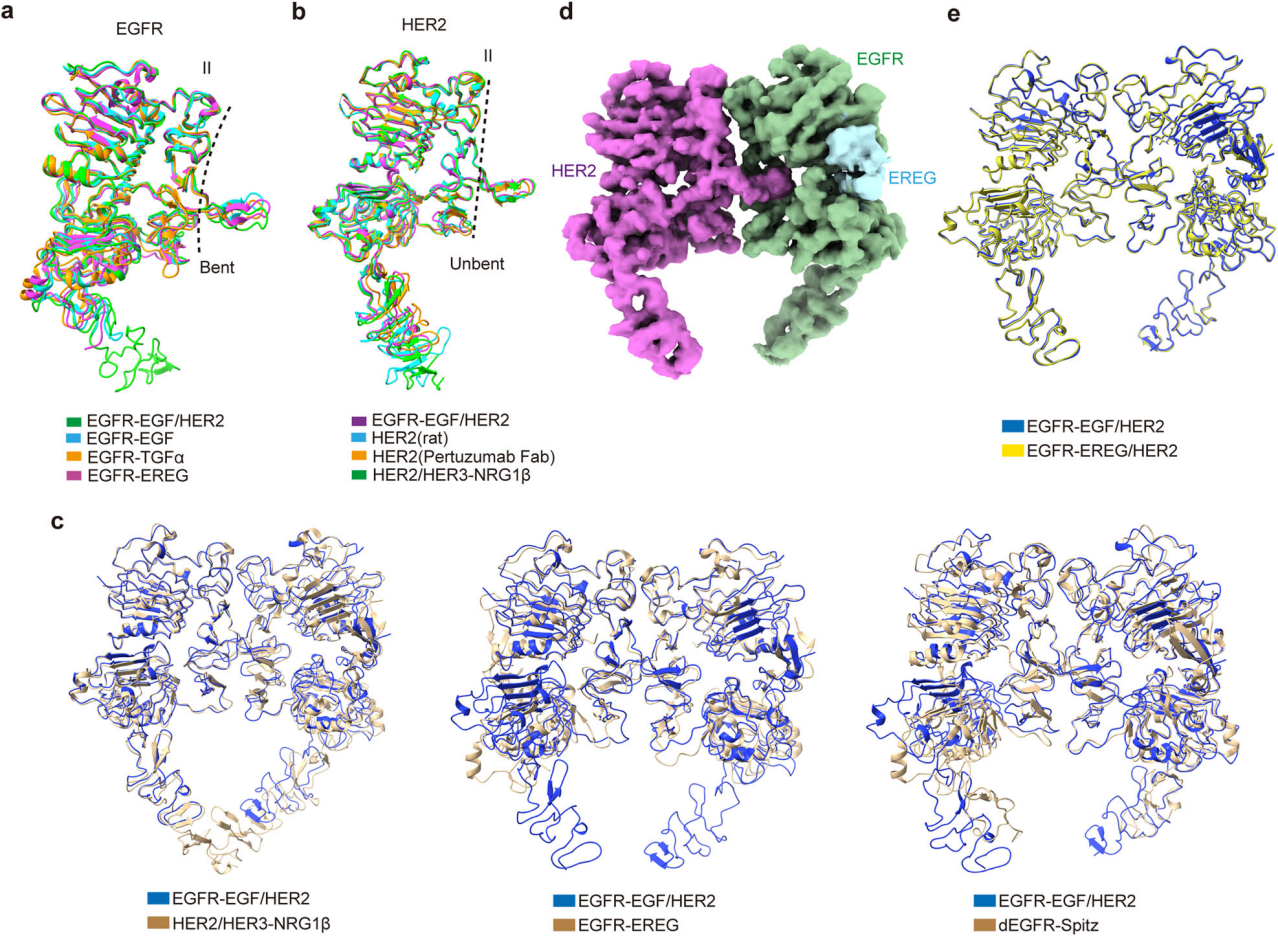
Structural comparison of different HER family proteins. **a** Superposition of the individual EGFR subunit structures in different dimers. EGFR in our EGFR/HER2 dimer (green) is superimposed with the bent EGFR protomer structures in EGF (cyan; PDB code: 1IVO)-, TGFα (orange; PDB code: 1MOX)-, and EREG (magenta; PDB code: 5WB7)-bound EGFR dimers. The RMSDs between our EGFR and the other three structures are 1.82 Å, 2.20 Å, and 1.72 Å, respectively. **b** Superposition of the individual HER2 subunit structures. HER2 in our EGFR/HER2 dimer (magenta) is overlaid with its monomer structure (rat) (cyan; PDB code: 1N8Y), as well as its structure in complex with Pertuzumab Fab (orange; PDB code: 1S78) or HER3 (green; PDB code: 7MN5). The RMSDs between our HER2 and the other three structures are 1.41 Å, 2.15 Å, and 1.34 Å, respectively. **c** Superposition of our EGFR/HER2 structure (blue) with the currently reported three asymmetric HER dimers (golden), namely, NRG1β-bound HER2/HER3 (left; PDB code: 7MN5; RMSD 1.95 Å), EREG-bound EGFR (middle; PDB code: 5WB7; RMSD 1.73 Å), and Spitz-bound *Drosophila* EGFR (dEGFR) (right; PDB code: 3LTG; RMSD 2.68 Å). **d** Cryo-EM map of the EREG-bound EGFR/HER2 ectodomain complex. EGFR, EREG, and HER2 are colored green, light blue, and magenta, respectively. **e** Superposition of the EGF (blue)- and EREG (yellow)-bound EGFR/HER2 structures.

The EGFR–EGF/HER2 dimer adopted a heart-shaped structure, similar to the dimer structures of other family members (Fig. 1d). EGF wedged into the cleft between Domains I and III of EGFR, stabilizing it in the extended conformation and exposing the dimerization interface of Domain II. Accordingly, Domain II of EGFR resembled the canonical bent conformation (Fig. 2a) seen in other dimer structures^15,16,18^. On the other side of the complex, HER2 retained the same conformation as in its monomer form or in complex with HER3 (Fig. 2b), consistent with its ligand-independent manner for signal transduction^23,30–32^. Since Domain II of HER2 was presented in the unbent conformation, it dimerized with EGFR in an asymmetric manner (Figs. 1c, d and 2c), consistent with that of the NRG1β-bound HER2/HER3, EREG-bound human EGFR, and Spitz-bound *Drosophila* EGFR complexes^17,18,23^. It is well known that different EGFR ligands induce distinct EGFR homodimer formations. Particularly, the high-affinity ligands EGF and TGFα stabilize the symmetric dimer, but the low-affinity ligand EREG generates the asymmetric one^15,16,18^. Thus, to verify whether different EGFR ligands might also affect the architecture of the EGFR/HER2 dimer, we further analyzed a 4.5 Å cryo-EM structure of the EREG-bound EGFR/HER2 complex (Fig. 2d; Supplementary Fig. S4 and Table S1). This structure is almost identical to the EGF-bound EGFR/HER2 complex, with a root-mean-square deviation (RMSD) of 0.63 Å (Fig. 2e), implying that the asymmetric EGFR/HER2 heterodimer is a common structure resulting from various EGFR ligands. Since the asymmetric EGFR homodimer has been shown to initiate a more sustained signal compared to the symmetric one^18^, the EGFR/HER2 heterodimer might exert its oncogenic effect through a similar mechanism to promote cell proliferation and transformation^33,34^.

### Unequal contribution of the DA for EGFR/HER2 assembly

**Fig. 3:**
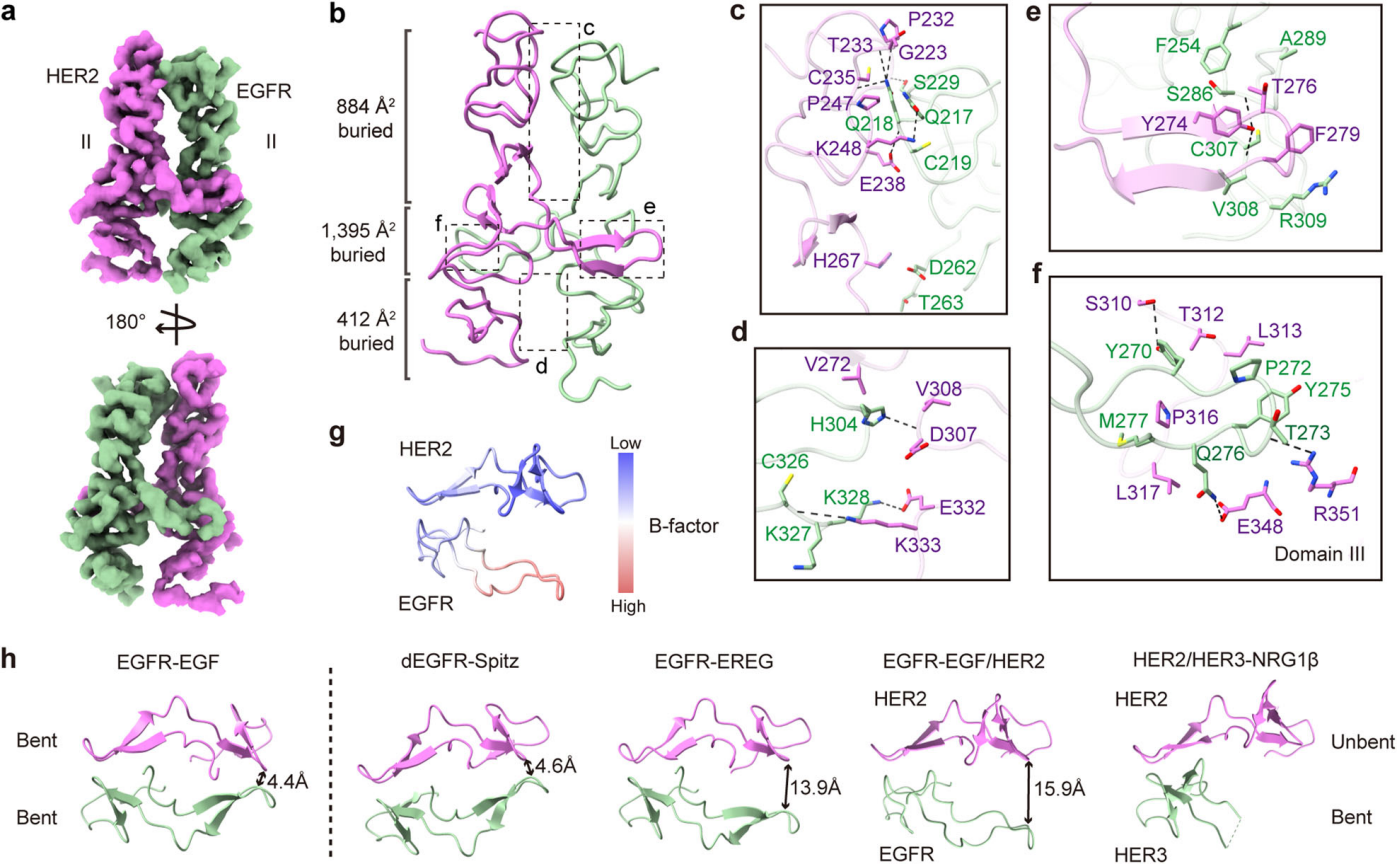
Interaction details between EGFR and HER2. **a** Cryo-EM map of the interface between Domain IIs of EGFR and HER2 shown in two views. **b** Structure of the EGFR/HER2 interface in ribbon presentation. EGFR is colored in green and HER2 is in magenta. The BSAs of different regions are indicated. **c**–**f** Interaction details between EGFR and HER2 as indicated in the insets of **b**. Residues involved in their interaction are shown with side chains. Black dashed lines represent hydrogen bond or salt bridge interactions (< 4.5 Å). **g** B-factor distribution of the DAs in the EGFR–EGF/HER2 structure. **h** Comparison of the structures of DAs in different HER dimers. From left to right: EGFR–EGF (PDB code: 1IVO), dEGFR–Spitz (PDB code: 3LTG), EGFR–EREG (PDB code: 5WB7), EGFR–EGF/HER2 (this study), and HER2/HER3–NRG-1β (PDB code: 7MN5). The DAs of the symmetric EGFR dimer exhibit the same conformation. For asymmetric dimers, the DAs of the unbent subunit pack closely with its counterpart, mimicking that of the symmetric EGFR dimer, whereas those of the bent subunit display various structures. The distances between DAs of the bent subunits and their partners are indicated.

Although the formation of HER family dimers is mainly mediated by Domain II, the interaction details are distinct for symmetric and asymmetric dimers^18^. The symmetric dimer relies heavily on DA-mediated contacts. Alternatively, the asymmetric dimer exhibits more interactions in the N-terminal region (Fig. 3a, b; Supplementary Fig. S5). Specifically, for the EGFR/HER2 complex, both the N- and C-terminal regions of Domain II interacted more closely with their counterparts relative to the EGFR homodimer, burying 884 Å^2^ and 412 Å^2^ surface areas, respectively (Fig. 3b–d). In comparison, the buried surface areas (BSAs) for the corresponding regions of the EGFR homodimer are only 535 Å^2^ and 327 Å^2^ (Supplementary Fig. S5). Strikingly, the DAs of EGFR and HER2 adopted different conformations. The DA of HER2 was inserted properly into its binding pocket on EGFR and made extensive polar and nonpolar interactions (Fig. 3e), whereas the tip region of the EGFR DA was almost dissociated from HER2, maintaining little contact with Domain III (Fig. 3f). Moreover, the EGFR DA was more flexible than HER2 DA, evident by its weaker cryo-EM densities and higher B-factor values (Fig. 3a, g). As a result, the total BSA between the DAs was only 1395 Å^2^ in the EGFR/HER2 complex (910 Å^2^ on the HER2 DA side and 485 Å^2^ on the EGFR DA side), which is less than that of the EGFR homodimer (1769 Å^2^) (Fig. 3b; Supplementary Fig. S5). Notably, in different asymmetric HER family dimers, the DA of the unbent subunit takes a similar conformation, fitting well into its binding pocket on the adjacent bent subunit (Fig. 3h); however, DAs of the bent subunits display diverse structures and loosely interact with the unbent subunit (Fig. 3h), indicating this bent arm might be insignificant for dimer formation. In fact, the bent subunit of the HER3 DA has already been shown to be unnecessary for its interaction with HER2^23^. Therefore, we wanted to investigate whether the DAs of EGFR and HER2 also contribute differently to their dimer assembly.

**Fig. 4:**
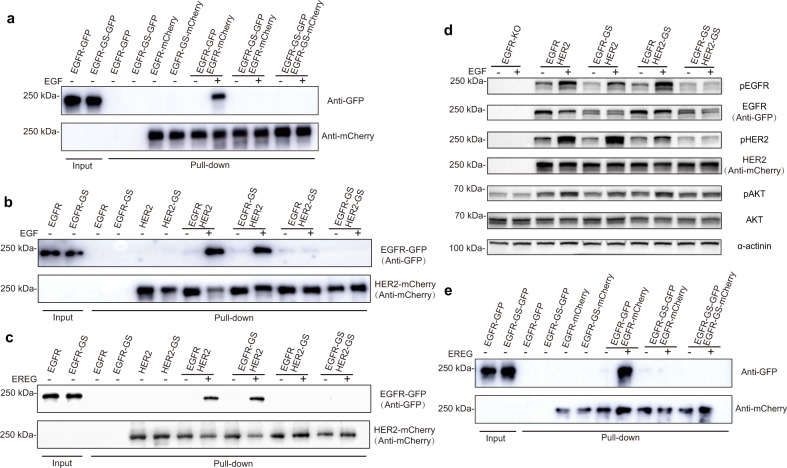
Functional significance of the DA of EGFR or HER2 for their dimer assembly. **a** Pull-down assay to verify the significance of DAs for EGF-induced EGFR dimerization. The mCherry-tagged EGFR was associated with the mCherry-nanobody (Nb) resin, and GFP-tagged EGFR was used as input for this assay. **b**, **c** Pull-down assay to verify the significance of DAs for EGF- (**b**) or EREG-induced (**c**) EGFR/HER2 interaction. The mCherry-tagged HER2 was associated with the mCherry-Nb resin, and GFP-tagged EGFR was used as input for this assay. **d** Detection of the phosphorylation levels of EGFR, HER2, and AKT upon EGF stimulation. Different EGFR and HER2 variants were transfected to the EGFR-knockout SUM159 cells. **e** Pull-down assay to verify the significance of DAs for EREG-induced EGFR dimerization.

To verify this point, we replaced the DA regions of full-length EGFR (residues 266–281) and tail-deleted HER2 (residues 270–285) with glycine–serine (GS) linkers (EGFR-GS and HER2-GS) and detected their interaction using a pull-down assay (Fig. 4). Our results showed that both DAs of individual EGFR protomers were crucial for EGF-induced EGFR dimer formation (Fig. 4a), consistent with their symmetric organization (Fig. 3h). However, for EGF- or EREG-induced asymmetric EGFR/HER2 dimer assembly, only the unbent subunit of the HER2 DA is required, but the bent subunit of the EGFR DA is dispensable (Fig. 4b, c). Next, we examined the impact of these EGFR and HER2 variants on downstream signaling. To rule out the influence of endogenous receptors, we performed the experiment in the EGFR knockout SUM159 human triple-negative breast cancer cell line, which has very low HER2 expression (Supplementary Fig. S6a, b). We coexpressed different variants of full-length EGFR and HER2 in these cells; upon EGF stimulation, HER2-GS, but not EGFR-GS, significantly reduced the phosphorylation of HER2 (Fig. 4d). Since the phosphorylation of HER2 depends solely upon its heterodimerization with EGFR, these results further confirmed that only the HER2 DA is important for the EGFR/HER2 heterodimer assembly and downstream signal transduction. Although EGFR-GS did not affect the phosphorylation of HER2, it severely disrupted the phosphorylation of EGFR (Fig. 4d), consistent with its critical role in EGFR homodimer formation^15,35,36^.

The structures of the two DAs in asymmetric HER family homodimers — namely, the EREG-bound human EGFR and Spitz-bound *Drosophila* EGFR — are also distinct (Fig. 3h), mimicking that of the heterodimers^17,18^. To investigate the role of the DA in asymmetric homodimer assembly, we analyzed the EREG-induced EGFR dimerization using a pull-down assay. We found that replacing one DA of EGFR with GS linkers drastically abolished the dimerization of EGFR (Fig. 4e), similar to that of the EGF-induced EGFR homodimerization (Fig. 4a), but different from the EGFR/HER2 (Fig. 4b, c) and HER2/HER3 heterodimerization^23^. This result suggested that the unequal contribution of DAs is only a factor in HER2-containing asymmetric dimer assembly, but not in asymmetric EGFR dimer formation. In fact, in the asymmetric HER family dimers, the relative distances between the DA of the bent subunit and its counterparts are different, such that the HER2-containing dimers are much further away (Fig. 3h). Therefore, from a structural point of view, it makes sense that the EGFR or HER3 DAs contribute minimally to their interaction with HER2.

### Single-molecule dynamics and interactions of endogenous EGFR and HER2 at the plasma membrane

**Fig. 5:**
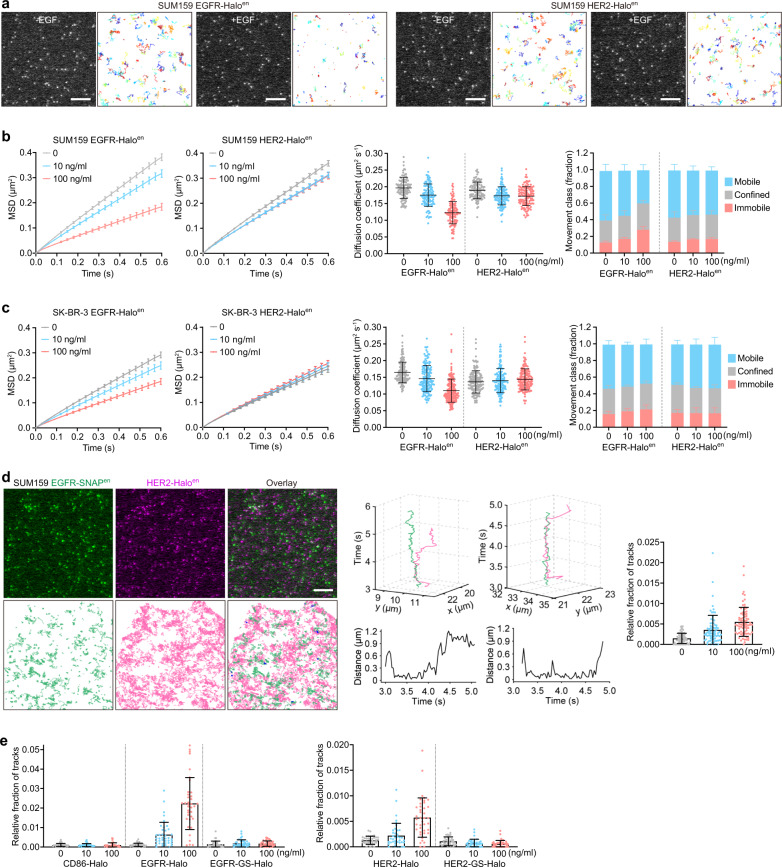
Single-molecule analyses of the diffusion dynamics and interactions of endogenous EGFR and HER2 in cancer cells. **a** Live-cell single-molecule imaging and tracking of endogenous (en) EGFR-Halo and HER2-Halo molecules at the plasma membrane of SUM159 cells genome-edited for EGFR-Halo/HER2-mEGFP or HER2-Halo/EGFR-mEGFP (labeled by JFX_650_-HaloTag ligand). Shown are representative single frames and tracking traces of time-lapse series acquired in the cells treated without or with EGF (100 ng/mL) by TIRF microscopy. **b** MSD-Δt plots (left two panels) and diffusion coefficients (middle panel) of EGFR-Halo tracks (*n* = 115, 109, and 110 cells from 4 independent experiments) and HER2-Halo tracks (*n* = 117, 119, 120 cells from four independent experiments) from genome-edited SUM159 cells treated with 0, 10, or 100 ng/mL EGF and imaged by TIRF microscopy. The right panel shows fractions of tracks classified as mobile, confined, or immobile (*n* = 4 independent experiments). **c** SK-BR-3 cells genome-edited for EGFR-Halo or HER-Halo were labeled by JFX_650_-HaloTag ligand, treated with 0, 10, or 100 ng/mL EGF, and imaged by TIRF microscopy. MSD-Δt plots (left two panels) and diffusion coefficients (middle panel) of EGFR-Halo tracks (*n* = 150, 156, and 158 cells from 4 independent experiments) or HER-Halo tracks (*n* = 152, 149, 153 cells from 4 independent experiments), and fractions of tracks classified as mobile, confined, or immobile (right panel, *n* = 4 independent experiments) are shown. **d** SUM159 cells genome-edited for both EGFR-SNAP and HER2-Halo were labeled by JFX_650_-SNAP-tag ligand and JFX_549_-HaloTag ligand, treated without or with EGF, and then imaged by TIRF microscopy. The representative single frame and tracking traces (co-localized trajectories are highlighted in blue) of the time-lapse series of a cell treated with 100 ng/mL EGF were shown on the left. Individual 3D trajectories (top) and distances (bottom) between EGFR-SNAP and HER2-Halo as a function of time are shown in the middle panels. The relative fractions of HER2 tracks that interact with EGFR in cells treated with 0, 10, or 100 ng/mL EGF are shown on the right (*n* = 73, 77, and 77 cells from 2 independent experiments). **e** SUM159 cells genome-edited for EGFR-SNAP were transiently expressed with HaloTag-tagged CD86, HER2, HER2-GS, EGFR, and EGFR-GS, labeled by JFX_650_-SNAP-tag ligand and JFX_549_-HaloTag ligand, and then imaged by TIRF microscopy. Shown are the relative fractions of tracks in Halo channels that interact with the SNAP-tagged endogenous EGFR (*n* = 36–40 cells from 2 independent experiments). Scale bars, 5 μm. Error bars show means ± SD except for the MSD-Δt plots (means ± 95% confidence interval).

Given that the EGFR/HER2 heterodimer is relatively unstable in our biochemical assays (Fig. 1a), it is intriguing to investigate their dynamics and interaction in live cells. The cancer-associated alterations of HER2 are more frequently related to its amplification and overexpression^2,37,38^; thus, we decided to comparatively characterize the membrane dynamics of endogenous EGFR and HER2 in two human breast cancer cell lines with either very low or very high HER2 expression — SUM159^39^ or SK-BR-3^40^, respectively (Supplementary Fig. S6a). We first employed the CRISPR/Cas9 genome-editing tool to fuse HaloTag to the endogenous EGFR or HER2 (EGFR-Halo or HER2-Halo) (Supplementary Fig. S6), labeled the cells with bright and photostable Janelia Fluor dyes (JFX dyes)^41^, and tracked the diffusion dynamics of stochastically labeled EGFR-Halo spots (0.16 ± 0.02 and 0.19 ± 0.04 fluorescent spots per μm^2^ on SUM159 and SK-BR-3 cells, respectively) or HER2-Halo spots (0.17 ± 0.02 and 0.18 ± 0.04 fluorescent spots per μm^2^ on SUM159 and SK-BR-3 cells, respectively) at a fast imaging rate (33 Hz) using TIRF microscopy (Fig. 5a; Supplementary Fig. S7a and Videos S1–S4). Automatic single particle detection and tracking revealed that most of the EGFR molecules exhibited free or mobile diffusion at the plasma membranes of both SUM159 and SK-BR-3 cells (Fig. 5a–c; Supplementary Fig. S7a and Videos S1–S4). Photobleaching of stochastically labeled EGFR-Halo or HER2-Halo spots showed that they were mainly single molecules (Supplementary Fig. S8a, b). Consistent with the imaging results obtained from other cell lines^42–44^, EGF stimulation, especially at high concentrations, decreased the diffusion rates of EGFR molecules in SUM159 and SK-BR-3 cells, as shown by the mean square displacement (MSD) and diffusion coefficient (*D*) analyses (Fig. 5b, c). In contrast, the diffusive behaviors of endogenous HER2 in these two cells were not significantly affected by EGF treatment (Fig. 5b, c). The different dynamics of EGFR and HER2 molecules at the plasma membrane upon EGF activation implied that the EGFR/HER2 heterodimer might not be stable within the cells as well.

Next, we sought to directly visualize the interplay between these two receptors. Although different biochemical or imaging-based methods have been used to detect the interaction between activated EGFR and HER2 in cell lysates or fixed cells^40,45^, the direct recording of EGFR/HER2 interaction in live cells has been technically challenging. By creating the genome-edited SUM159 cells expressing both EGFR-SNAP and HER2-Halo (Supplementary Fig. S6d), labeling the cells with the JFX_549_-HaloTag ligand (0.31 ± 0.09 fluorescent spots per μm^2^) and JFX_650_-SNAP-tag ligand (0.24 ± 0.11 fluorescent spots per μm^2^), and imaging the cells with two-color single-molecule TIRF microscopy, we achieved the direct visualization and tracking of the interaction between stochastically labeled endogenous EGFR and HER2 molecules (Fig. 5d). The reduced or largely unchanged diffusion dynamics of EGFR and HER2 were also observed when they were simultaneously labeled in the same cells (Fig. 5d; Supplementary Fig. S7b). Notably, we found that EGF readily induced the transient interaction of EGFR and HER2 molecules in a concentration-dependent manner (Fig. 5d), which was also visualized when HER2-Halo was transiently expressed in the genome-edited SUM159 cells expressing EGFR-SNAP (Fig. 5e). As negative controls, HER2-GS and a non-related membrane receptor CD86^46^ did not interact with EGFR upon EGF stimulation (Fig. 5e). Collectively, these results provide direct evidence for the existence of EGFR/HER2 dimers on the plasma membrane of live cells and indicate that the specific transient interaction between activated EGFR and HER2 indeed relies on the DA of HER2, in support of our structural and biochemical studies.

### Interaction with HER2 impedes the endocytosis of EGFR

**Fig. 6:**
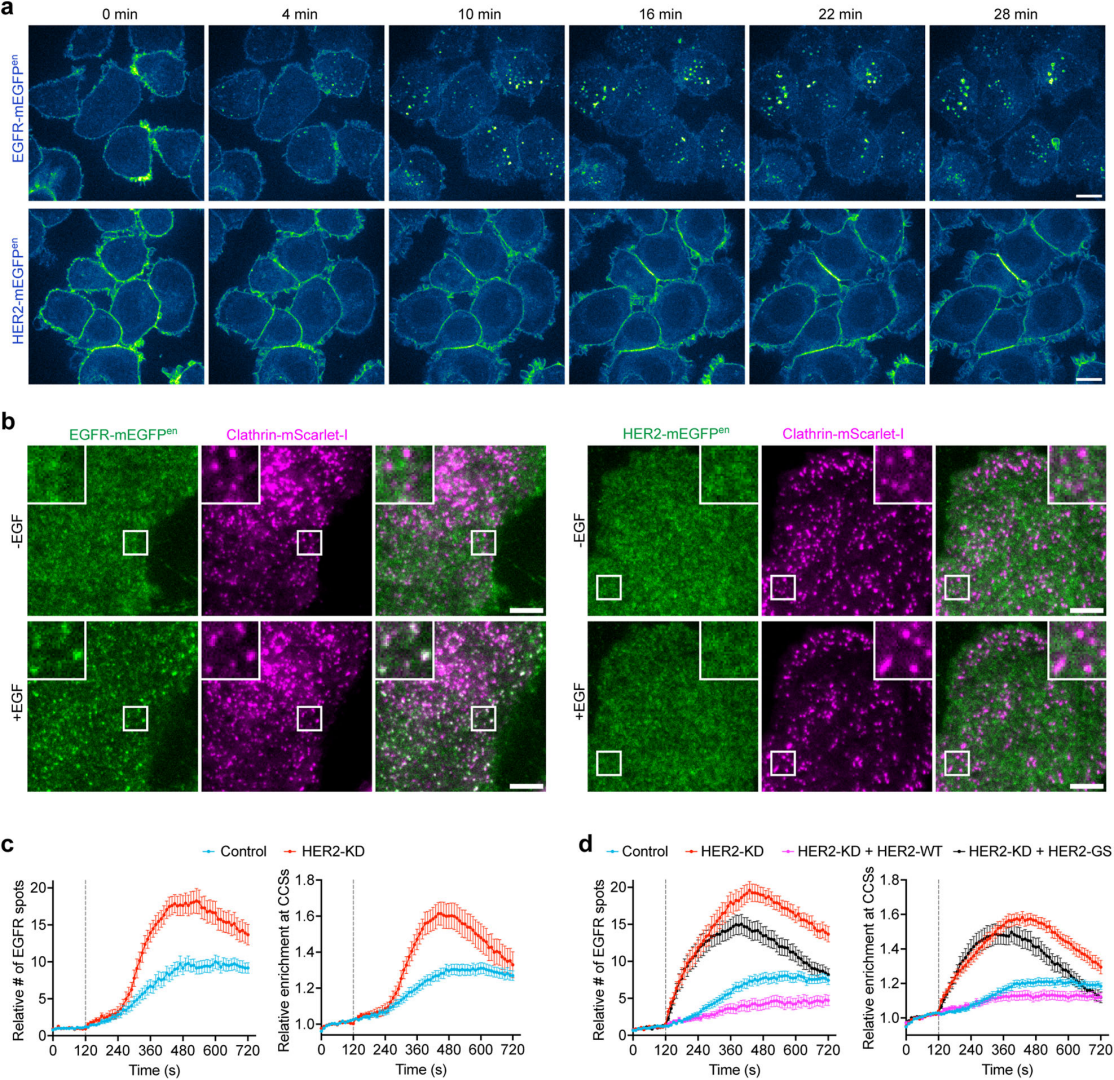
Interaction of HER2 and EGFR inhibited EGFR endocytosis. **a** SK-BR-3 cells genome-edited for EGFR-mEGFP or HER2-mEGFP were imaged by spinning-disk confocal microscopy. Shown are images of the cells in the middle planes at indicated times after EGF (100 ng/mL) treatment. Scale bars, 10 μm. **b** SK-BR-3 cells genome-edited for EGFR-mEGFP or HER2-mEGFP stably expressing clathrin-mScarlet-I were imaged at the bottom surfaces by TIRF microscopy. Shown are the single frames before and 3 min after EGF treatment during the continuous time-lapse imaging. Boxed regions are enlarged and shown. Scale bars, 5 μm. **c** SK-BR-3 cells genome-edited for EGFR-mEGFP and stably expressing clathrin-mScarlet-I were treated with control siRNA or siRNA targeting HER2 (HER2-KD), and then imaged at the bottom surfaces by TIRF microscopy. EGF was added at 120 s of the time-lapse imaging. The relative numbers of fluorescence spots of EGFR-mEGFP that appeared at the plasma membrane (left panel) and the relative enrichment of EGFR-mEGFP fluorescence in clathrin-coated structures (CCSs, right panel) during EGF stimulation are shown (*n* = 23 and 19 cells). **d** SK-BR-3 cells genome-edited for EGFR-mEGFP and stably expressing clathrin-mScarlet-I were treated with control siRNA or siRNA targeting HER2 (HER2-KD). The cells treated with siRNA targeting HER2 were transiently transfected with the siRNA-resistant wild-type HER2 or the HER2-GS mutant and then imaged at the bottom surfaces by TIRF microscopy. EGF was added at 120 s of the time-lapse imaging. The relative numbers of fluorescence spots of EGFR-mEGFP that appeared at the plasma membrane (left panel, *n* = 49, 50, 29, and 30 cells) and the relative enrichment of EGFR-mEGFP fluorescence in CCSs (right panel, *n* = 43, 49, 28, and 30 cells) during EGF stimulation are shown. Error bars show means ± SEM.

Ligand binding to EGFR induces the assembly and subsequent endocytosis of the signaling complexes^47–51^, which causes the reduced mobility of receptors at the plasma membrane and is crucial for signal attenuation^42,43,52^. The largely diffusive motion of HER2 upon EGF activation suggests that it does not undergo endocytosis either on its own or in complex with EGFR, as documented in previous studies with ectopically overexpressed HER2^7,53^. To corroborate this finding with our live-cell imaging system, we used spinning disk confocal microscopy to track in real-time the fate of activated endogenous HER2 and EGFR in genome-edited SK-BR-3 cells expressing HER2-mEGFP or EGFR-mEGFP. Whereas EGFR was internalized and started to accumulate inside the cells soon after EGF stimulation (< 5 min), HER2 remained on the plasma membrane for a long period (> 20 min), even at the high EGF concentration of 100 ng/mL (Fig. 6a). By using the more sensitive TIRF microscopy, we further confirmed that EGF could effectively induce the oligomerization and accumulation of endogenous EGFR, but not HER2, in the clathrin-coated endocytic structures in SK-BR-3 cells (Fig. 6b; Supplementary Fig. S8c, d). These results are consistent with our observations that EGF stimulation did not slow down the single molecule movement of HER2 at the plasma membrane (Fig. 5b, c).

Since HER2 can resist endocytosis, we investigated whether it would further influence the endocytosis of EGFR through their interaction. Therefore, we knocked down the expression of HER2 (HER2-KD) in SK-BR-3 cells and used TIRF microscopy to track in real-time the endocytosis process of endogenous EGFR. Our results showed that decreasing the expression of HER2 significantly accelerated EGFR oligomerization and enrichment in clathrin-coated structures (Fig. 6c), which could be rescued by overexpressing the wild-type HER2 but not the EGFR binding-defective HER2-GS variant (Fig. 6d). The uptake of fluorescently labeled EGF was also increased in SK-BR-3 cells with HER2-KD (Supplementary Fig. S8e). Thus, not only can HER2 resist endocytosis individually, but — through the formation of transient heterodimers — it can also help another HER family member EGFR escape internalization and downregulation.

## Discussion

It was identified over three decades ago that EGFR and HER2 associate with each other and work synergistically to mediate the cell transformation^7–11^. However, the biochemical details of their interaction have remained unclear until this work. Like the previously reported asymmetric HER dimer structures^17,18,23^, the EGFR/HER2 structure that we resolved provides more evidence to demonstrate that asymmetric ectodomain dimerization is an important functional state for HER family members. For symmetric EGFR dimers, Tyr275 in the DA and Arg309 in the binding pocket of its partner make key intermolecular cation–π interactions to stabilize the complex^15,16^. Notably, these two residues become phenylalanine (Phe279) and leucine (Leu313) in HER2, respectively (Fig. 3e, f). Structurally, the cation–π interaction is largely unaffected by the tyrosine-to-phenylalanine mutation, but is destroyed by converting the arginine to leucine. Therefore, in HER2-containing dimers, it is likely that only the DA of HER2 can insert appropriately into the binding pocket of its partner, but not conversely. As a result, HER2 could only form asymmetric dimers with other family members as observed in the EGFR/HER2 and HER2/HER3 structures^23^. Furthermore, our functional studies demonstrated that only the well-placed DA of HER2, but not that of EGFR, is required for their dimerization, consistent with the structural features that we observed.

It has been well accepted that the carcinogenesis of HER2 is due to the more prolonged and/or enhanced signaling of HER2-containing heterodimers compared to the homodimers of other family members^54,55^. Specifically, in EGFR signaling, it has been reported that HER2 can extend the EGFR signaling duration either by attenuating EGFR endocytosis or shunting the internalized EGFR toward recycling and away from degradation^1,53,56,57^. However, the comparative characterization of the behaviors of endogenous EGFR and HER2 in live cells during EGF activation has not been achieved, which greatly limits our understanding of their intricate dynamics, interaction, and pathogenic effects. Here, we tracked and analyzed the membrane dynamics of endogenous EGFR and HER2 molecules using single-molecule live-cell imaging. We observe the transient interaction between activated EGFR and HER2 molecules at the cell membrane of cancer cells in real time. More importantly, we demonstrated that HER2 impeded the endocytosis of EGFR, which could lead to the prolonged downstream signaling seen in previous studies^53,56^. Surprisingly, HER2 does not perform this function through higher affinity to EGFR, which would competitively disrupt EGFR homodimerization and internalization. On the contrary, we found that the interaction between HER2 and EGFR is quite weak and short-lived. In situations wherein HER2 is highly overexpressed at the plasma membrane of certain cancer cells, the accumulation of transient interactions exerted by large numbers of HER2 molecules is likely sufficient to influence the membrane dynamics and endocytosis of EGFR. This mechanism of interaction could provide an advantage in cancer cells; since the dynamics and thus concentrations of HER2 itself at the plasma membrane would not be affected, this would ensure that HER2 can continuously impose the regulatory effects on EGFR. Furthermore, HER2 might also regulate the function of other family members in a similar manner. Overall, this work provides elaborate details on the dynamics and interaction of EGFR and HER2, and deepens our understanding of the EGFR/HER2 signaling.

## Materials and methods

### Cell cultures

*Sf*9 insect cells (ATCC CRL-1711) were cultured at 27 °C in Sf-900 II SFM medium (Gibco). HEK293S GnTI^–^ cells (ATCC CRL-3022) were cultured at 37 °C with 5% CO_2_ in Yocon HEK293 medium (Yocon Biotechnology), supplemented with 1% fetal bovine serum (FBS) and 100 μg/mL penicillin/streptomycin (Gibco). SUM159 cells were cultured at 37 °C and 5% CO_2_ in DMEM/F-12 (Corning), supplemented with 5% FBS (Gibco), 100 U/mL penicillin and streptomycin (Corning), 1 μg/mL hydrocortisone (Sigma-Aldrich), 5 μg/mL insulin (Sigma-Aldrich), and 10 mM HEPES (Corning), pH 7.4. SK-BR-3 cells were cultured at 37 °C and 5% CO_2_ in RPMI 1640 (Gibco), supplemented with 10% FBS (Gibco) and 100 U/mL penicillin and streptomycin (Corning). The cells were routinely verified to be mycoplasma-free using the TransDetect PCR Mycoplasma Detection Kit (TransGen Biotech). The *Escherichia coli* TransB cells (TransGen Biotech, TRANS CD811-02) were cultured at 37 °C in Luria-Bertani medium with 50 mg/L kanamycin and 10 mg/L tetracycline.

### Expression and purification of EGF

A gene encoding the 53 amino-acid of human EGF (NSDSECPLSHDGYCLHDGVCMYIEALDKYACNCVVGYIGERCQYRDLKWWELR) was synthesized and cloned into a modified pET-32a vector (Novagen) which bears an N-terminal 6× histidine + SUMO tag followed by a PreScission protease recognition site. This plasmid was transformed into *E. coli* (TransB) for protein expression. Bacteria were grown in 1 L of Luria-Bertani medium at 37 °C with 50 mg/L kanamycin, 10 mg/L tetracycline, and 100 mg/L ampicillin. When OD_600_ reached 1.0, the protein expression was induced at 20 °C by adding isopropyl β-D-thiogalactoside (IPTG) to a final concentration of 0.5 mM. After 24 h, the cells were centrifuged at 7000 rpm for 20 min and frozen at –80 °C.

For the purification of EGF, cells were thawed and broken by sonication in PBS buffer. The cell debris was removed by centrifugation at 18,000 rpm for 40 min. Subsequently, the supernatants were mixed with Ni NTA beads (Smart-Lifesciences) at 4 °C for 1.5 h in the presence of 5 mM imidazole. The beads were washed with 30 column volumes (CVs) of PBS buffer containing 10 mM imidazole, and the protein was eluted using PBS buffer supplemented with 250 mM imidazole. The 6× histidine + SUMO tag was cleaved by addition of PreScission protease (8:1 w/w ratio). After dialyzed overnight in PBS buffer, the sample was re-incubated with nickel beads to remove the tag. The flow-through fraction was further purified by gel filtration using a Superose 6 Increase 10/300 GL column (GE Healthcare) equilibrated with PBS buffer. The peak fractions of the target protein were collected, flash frozen in liquid nitrogen, and stored at –80 °C.

### Expression and purification of the EGFR/HER2 complex

DNA sequence of human EGFR (residues 1–683, EGFR_JM) with a C-terminal basic coiled-coil peptide (AQLKKKLQALKKKNAQLKWKLQALKKKLAQ) was cloned into a pEG BacMam expression vector^58^ bearing a green fluorescent protein (GFP) tag at the C-terminus. The sequence of human HER2 (residues 1–693, HER2_JM) with a C-terminal acidic coiled-coil peptide (AQLEKELQALEKENAQLEWELQALEKELAQ) was cloned into another vector carrying a 10× histidine + mCherry tag at the C-terminus. Additionally, a TEV protease cleavage site surrounded by two GGS linkers (GGSENLYFQGGGS) was inserted between the receptors and coiled-coil peptides. These plasmids were transformed into DH10Bac *E. coli* cells to generate bacmids, and then the recombinant baculoviruses were produced in *Sf*9 cells using Cellfectin II reagents (Life Technologies). HEK293S GnTI^–^ suspension cells at a density of 3 × 10^6^ cells/mL were co-infected with 5% passage 3 viruses of both EGFR and HER2. The cells were first cultured at 37 °C for 8–12 h, and then transferred to 30 °C for 48 h with addition of 10 mM sodium butyrate. Cells were harvested by centrifugation at 6000 rpm for 40 min, and stored at –80 °C.

For the purification of EGFR/HER2 complex, cells were resuspended in Buffer A (50 mM HEPES, pH 7.5, 150 mM NaCl, 15% glycerol, 2 μg/mL DNase, and a cocktail of protease inhibitors (1 μg/mL Aprotinin, 1 μg/mL Leupeptin, 1 μg/mL Pepstatin, 20 μg/mL Trypsin inhibitor, 1 mM Benzamidine, and 1 mM PMSF)). Before solubilized with 1% *n*-dodecyl-β-d-maltoside (DDM, Anatrace) and 0.1% cholesteryl hemisuccinate (CHS, Anatrace) at 4 °C for 2 h, the cells were incubated with 0.2 μM EGF or EREG (Beyotime) for 1 h. Then the supernatants were separated by centrifugation at 18,000 rpm for 40 min, and mixed with Ni NTA beads at 4 °C for 1.5 h. The nickel beads were rinsed sequentially with 30 CVs of Buffer B (25 mM HEPES, pH 7.5, 150 mM NaCl, and 0.02% DDM-0.002% CHS) containing 10 mM and 50 mM imidazole. The proteins were eluted using Buffer B supplemented with 250 mM imidazole. Next, the sample was applied to anti-GFP nanobody (GFPnb)-coupled CNBR-activated Sepharose beads (GE Healthcare). After mixing at 4 °C for 2 h, the beads were washed with 20 CVs of Buffer B, and then incubated with PreScission protease (4:1 w/w ratio) at 4 °C overnight to release the target proteins. The proteins were loaded into a Superose 6 Increase 10/300 GL column for further purification. Only fresh-purified proteins were used for the following cryo-EM studies.

### Cryo-EM sample preparation, data collection, and processing

The EGF- and EREG-bound EGFR/HER2 complexes from the peak fractions of gel filtration were concentrated to 4.2 mg/mL. A total of 3 μL samples were deposited to Quantifoil R1.2/1.3 300 Au holey carbon grids (Quantifoil), and then blotted and frozen in liquid ethane using Vitrobot Mark IV (FEI). The grids were stored in liquid nitrogen until further use. Data collection was performed using a 300-kV Titan Krios (FEI) microscope equipped with a K3 summit detector (Gatan) and a Gatan imaging filter (GIF) with a slit of 20 eV. SerialEM software^59^ was used for automatic data acquisition. The data were collected in super-resolution mode at a physical pixel size of 1.07 Å. The dose rate was 14.5 e^–^/pixel/s and total exposure time was 4.8 s. Each micrograph was divided into 40 frames. The total number of micrographs collected for the EGF- and EREG-bound EGFR/HER2 complexes was 6121 and 8067, respectively.

The data processing strategy for these two datasets was the same. Raw images were motion-corrected using MotionCor2^60^. Contrast transfer function (CTF) parameters for each micrograph were calculated using Gctf^61^. Particle picking, two-dimensional (2D) and three-dimensional (3D) classifications, and 3D refinement were performed in RELION-3.1^62^. For both datasets, the good subclasses out of 2D classification were used for the following 3D classification. For the EGF-bound EGFR/HER2 dataset, the EGFR/HER2 heterodimer and EGFR homodimer were classified into two different 3D classes with nearly equal particle amounts, both of which were applied to 3D refinement. For the EREG-bound EGFR/HER2 dataset, only one of the 3D classes with the highest resolution representing the EGFR/HER2 heterodimer was chosen for 3D refinement. After 3 rounds of alternative Bayesian polishing and CTF refinement, the particle stacks were imported into cryoSPARC^63^ and subjected to non-uniform and local refinement. The local resolution distribution of the final maps was estimated in cryoSPARC, and DeepEMhancer^64^ was used for post-processing. The resolutions of the EGF-bound EGFR/HER2 and homodimeric EGFR, and EREG-bound EGFR/HER2 complexes were 3.3 Å, 3.8 Å, and 4.5 Å, respectively (using the 0.143 cutoff criterion).

### Model building and refinement

The reported structures of NRG1β-bound HER2/HER3 (PDB code: 7MN5) and EGF-bound EGFR (PDB codes: 1IVO and 7SYD) were used for the model building of our EGF-bound EGFR/HER2 and dimeric EGFR complexes. The corresponding structures were roughly fitted into our cryo-EM maps using ChimeraX^65^ and then manually adjusted using COOT^66^. The real-space refinement was performed in PHENIX^67^. For the model building of the EREG-bound EGFR/HER2 complex, the EGF-bound heterodimer structure was fitted into the EM map and EGF was subsequently substituted with EREG (PDB code: 5WB7). The following refinement was also carried out in PHENIX. The Fourier shell correlation (FSC) curves between the refined models and full maps were calculated using RELION. The final models were validated using MolProbity^68^. All the figures were created with ChimeraX.

### FSEC

DNA sequence of human EGFR (residues 1–1210) was cloned into a pEG BacMam vector with a C-terminal GFP tag. The sequence of human HER2_DT (residues 1–1029, C-terminal tail-deleted version) was cloned into another vector carrying a maltose-binding protein (MBP) in tandem with a mCherry tag at the C-terminus. The plasmids of EGFR and HER2_DT, or EGFR_JM and HER2_JM, were co-transfected into HEK293S GnTI^–^ cells. Specifically, 0.5 μg of each plasmid was mixed with 3 μg PEI MAX 40K (Poly Sciences) and incubated in 200 μL OPT-MEM (Gibco) medium at room temperature for 25 min before adding into 2 mL adherent cells. The cells were cultured at 37 °C for 8–12 h, and then transferred to 30 °C for 48 h with addition of 10 mM sodium butyrate. Cells were harvested by centrifugation at 4000 rpm for 5 min and subsequently incubated with 5 μM EGF for 1 h in Buffer A. Next, the cells were solubilized with 1% DDM and 0.1% CHS at 4 °C for 2 h. The supernatants were separated by centrifugation at 15,000 rpm for 1 h and loaded into a Shimadzu HPLC system. The proteins were separated by a Superose 6 Increase 10/300 GL column during which process the GFP and mCherry fluorescence signals were monitored.

### Pull-down assays

A total of 6× histidine + GFP-tagged EGFR and EGFR-GS were purified with Ni NTA beads. mCherry-tagged HER2_DT and HER2-GS_DT were attached to anti-mCherry nanobody (mCherrynb)-coupled beads in Buffer B. In total 10 μL beads were mixed with 28 μg EGFR or EGFR-GS at 4 °C for 2 h in the presence of 10 μM EGF or 100 μM EREG. After washing three times with Buffer B, the beads were added to the SDS loading buffer and boiled at 100 °C for 8 min. The protein extracts were separated using 10% SDS polyacrylamide gel and transferred onto polyvinylidene fluoride (PVDF) membranes. For the immunoblotting of GFP-tagged EGFR, the membranes were blocked at room temperature for 1 h and then incubated with mouse anti-GFP primary antibody (TransGen Biotech, 1:5000) at 4 °C overnight in TBST buffer (TBS + 1% Tween) supplemented with 5% skim milk. Subsequently, the membranes were incubated with goat anti-mouse IgG secondary antibody (TransGen Biotech, 1:10,000) at room temperature for 1 h. The signal was detected using a High-sig ECL Western Blotting Substrate (Thermo Fisher) and the images were captured using a AI 600 (Cytiva) equipment. For probing mCherry-tagged HER2 with the same membrane, stripping and re‐probing were performed. Briefly, the PVDF membranes were incubated with the stripping buffer (62.5 mM Tris-HCl, 2% SDS, and 100 mM β-mercaptoethanol) at room temperature for 30 min. After washing three times with TBST, the membranes were sequentially incubated with mouse anti-mCherry primary antibody (Beyotime, 1:10,000) and goat anti-mouse secondary antibody. The signals were redetected in the same way as above.

### Immunoblotting analyses for protein phosphorylation detection

The EGFR knockout SUM159 cells were co-transfected with the plasmids expressing wild-type or mutant EGFR (EGFR-mEGFP or EGFR-GS-mEGFP) and HER2 (HER2-mCherry or HER2-GS-mCherry) for 8 h, and then serum-starved for 16 h. Next, the cells were treated with EGF (10 ng/mL, Peprotech) for 10 min, washed with DPBS once, and solubilized at 4 °C for 30 min in RIPA lysis buffer (Sigma-Aldrich) with a protease inhibitor cocktail (Pierce). The samples were pelleted at 13,400× *g* for 15 min at 4 °C, and then mixed with 5× sample buffer (GenScript) and heated to 100 °C for 10 min. After fractionated by 10% SDS–PAGE, the proteins were transferred to nitrocellulose membranes (Cell Signaling). The membranes were incubated in TBST buffer containing 5% skim milk for 3 h at room temperature, followed by overnight incubation at 4 °C with the specific primary antibodies. After three washes in TBST (5 min each), the membranes were incubated with the appropriate HRP-conjugated secondary antibody (Beyotime, 1:1000) at room temperature for 1 h. After three washes (5 min each) in TBST, the membrane was incubated with the SignalFire^TM^ ECL Reagent (Cell Signaling) and imaged by the Tanon-5200 Chemiluminescent Imaging System (Tanon). The primary antibodies agonist AKT (4691S, 1:1000, Cell Signaling), α-actinin (69758S, 1:1000, Cell Signaling), phospho-AKT (4060S, 1:2000, Cell Signaling), phospho-EGFR (3777S, 1:1000, Cell Signaling), GFP (HT801-01, 1:3000, TransGen Biotech), mCherry (26765-1-AP, 1:3000, Proteintech), and phospho-HER2 (Y1221 + Y1222) (ab91633, 1:1000, Abcam) were used in this study.

### Plasmids and reagents for single-molecule imaging

The plasmids used for transient expression in mammalian cells (EGFR-mEGFP, EGFR-GS-mEGFP, EGFR-Halo, EGFR-GS-Halo, HER2-Halo, HER2-GS-Halo, CD86-Halo) were generated by the Gibson assembly method. A linker (5′-GGAGGTTCTGGTGGTTCTGGTGGTTCC-3′) was placed between EGFR/HER2 and mEGFP/Halo. Transfections were performed using Lipofectamine 3000 (Invitrogen) according to the manufacturer’s instructions.

JFX_650_-HaloTag ligand, JFX_549_-HaloTag ligand, and JFX_650_-SNAP-tag ligand were kind gifts from Luke D. Lavis (Janelia Research Campus). EGF was purchased from PeproTech.

### Incorporation of tags to EGFR or HER2 in SUM159 and SK-BR-3 cells using the CRISPR/Cas9 approach

SUM159 and SK-BR-3 cells were genome-edited to incorporate mEGFP, Halo, or SNAP tags to the C-terminus of EGFR or HER2 using the CRISPR/Cas9 approach as described^69,70^. The single-guide RNA (sgRNA) targeting human *EGFR* (5′-TTATTGGAGCATGACCACGG-3′) or human *HER2* (5′-CTTGGCCTTCTGGTTCACAC-3′) was cloned into pSpCas9(BB)-2A-Puro (PX459) (Addgene). The donor constructs EGFR-mEGFP, EGFR-Halo, EGFR-SNAP, HER2-mEGFP, and HER2-Halo used for homologous recombination were generated by cloning into the pUC19 vector with two ∼600–800-nucleotide fragments of genomic DNA upstream and downstream of the stop codon of human *EGFR* or *HER2*, and the open reading frame of mEGFP, Halo or SNAP using the pEASY-Uni Seamless Cloning and Assembly Kit (TransGen Biotech).

SUM159 and SK-BR-3 cells were transfected with the donor plasmid and sgRNA targeting sequence containing PX459 plasmid using Lipofectamine 3000 (Invitrogen) according to the manufacturer’s instruction. The cells expressing mEGFP, Halo (stained by JF_549_-HaloTag ligand), or SNAP (stained by JF_549_-SNAP-tag ligand) were enriched by fluorescence-activated cell sorting (FACS) (FACSAria II, BD Biosciences). SUM159 cells expressing EGFR-mEGFP, EGFR-Halo, or EGFR-SNAP were further subjected to single cell sorting to 96-well plates. The clonal SUM159 cells expressing EGFR-mEGFP^+/+^, EGFR-Halo^+/+^, or EGFR-SNAP^+/+^ were then gene-edited for HER2 to generate a pool of cells expressing EGFR-mEGFP HER2-Halo, EGFR-Halo HER2-mEGFP, and EGFR-SNAP HER2-Halo. SK-BR-3 cells expressing EGFR-mEGFP, EGFR-Halo, HER2-mEGFP, or HER2-Halo were subjected to two more subsequent bulk sorting to enrich the genome-edited cell pools. The genome-edited SUM159 and SK-BR-3 cells were confirmed by imaging, PCR, and western blot analysis.

### Knockout of EGFR in SUM159 cells

Knockout of EGFR in SUM159 cells was performed using the CRISPR/Cas9 approach as described^69,70^. The sgRNA sequence targeting the human *EGFR* gene is 5′-ATAGTTAGATAAGACTGCTA-3′. The monoclonal cell populations were screened by loss of EGFR protein expression by western blotting and confirmed by genomic DNA sequencing.

### Single-molecule imaging and analyses of the diffusive dynamics of EGFR and HER2

Single-molecule TIRF imaging was performed on a Nikon Ti2-E microscope equipped with a CFI Apochromat TIRF 100× objective (1.49 NA, Nikon), a manual TIRF Illuminator Unit (Nikon), a Perfect Focus Unit (Nikon), a UNO Stage Top Incubator (Okolab), and OBIS CellX 405, 488, 561, and 637 nm lasers (Coherent). The emission fluorescence signal was collected using the W-VIEW GEMINI-2C Image Splitting Optics (Hamamatsu) and two EMCCD cameras (Evolve 512 Delta, Photometrics). The 2× tube lens equipped on the Nikon Ti2-E microscope was applied for single-molecule imaging experiments (final pixel size corresponding to 80 nm of the image). Imaging sequences were acquired using Micro-Manager 1.4^71^.

Genome-edited SUM159 or SK-BR-3 cells expressing EGFR-Halo or HER2-Halo were cultured overnight in 4-well confocal dishes (Cellvis). The cells were then incubated with JFX_650_-HaloTag ligand in culture medium for 3–5 min, washed three times with culture medium, and then imaged in phenol-free DMEM/F12 (Corning) containing 5% FBS and 20 mM HEPES. The cells were then imaged by the single-molecule TIRF microscopy. Each cell was imaged continuously for 400 frames with an exposure time of 30 ms. The 150 to 300 frames of the time-lapse movies were used for imaging analysis. The images were subjected to cell mask creation by Fiji. Then the single fluorescent spots in the processed images were detected and tracked by u-track^72^ in MATLAB (MathWorks). The parameters with non-default values used: psfSigma, 1.25; minTrackLen, 4; minSearchRadius, 2; maxSearchRadius, 6; maximum allowed gaps, 4.

The tracks longer than 20 frames were used for mean square displacement (MSD) analysis using the MSDanalyzer package^73^ in MATLAB. The diffusion coefficients were calculated using the first five points with a minimal fitting R^2^ value of 0.8 using the MSDanalyzer package^73^. The tracks were classified as mobile, confined, immobile, and directed based on the moment scaling spectrum of each track by u-track^72^. Since the fraction of tracks classified as directed was less than 3%, the tracks classified as mobile, confined, and immobile were used for further analysis.

### Single-molecule imaging and analyses of the interaction between EGFR and HER2

Genome-edited SUM159 cells expressing both EGFR-SNAP and HER2-Halo were cultured overnight in the pretreated confocal dishes (Cellvis). The confocal dishes were sonicated in NaOH for 1 h, washed with distilled H_2_O three times, and then stored in 100% ethanol. The cleaned dishes were washed three times with DPBS before use. Cells cultured overnight on the cleaned dished were then incubated with a mixture of JFX_549_-HaloTag ligand and JFX_650_-SNAP-tag ligand in culture medium for 5 min, washed three times with culture medium, and then imaged in phenol-free DMEM/F12 containing 5% FBS and 20 mM HEPES. Single-molecule dual color imaging was performed with the same TIRF microscopy described above. JFX549 and JFX650 were excited and imaged sequentially for 400 frames with an exposure time of 30 ms. The 100 to 400 frames of the dual-color movies were subjected to cell mask creation by Fiji, and then detected and tracked by u-track for each channel^72^. The parameters with non-default values used: psfSigma, 1.25; minTrackLen, 4; minSearchRadius, 2; maxSearchRadius, 8; maximum allowed gaps, 4. For colocalization analysis, the two channels were firstly aligned by the 0.1-μm multi-color TetraSpeck beads (Thermo Fisher) using a transformation matrix from the software package SlimFast4C^74,75^. A non-related membrane receptor CD86^46^ was used as a reference for random colocalization. Molecules within tracks from the two channels that fall within a 100 nm radius were identified as colocalized by the software package SlimFast4C^74,75^. Trajectories with at least five consecutive colocalized frames were identified as co-locomotion.

### Real-time tracking of EGFR and HER2 endocytosis by TIRF and spinning disk confocal microscopy

The endocytosis process at the plasma membrane was tracked with a Nikon TIE microscope equipped with a CFI Apochromat TIRF 100× objective (1.49 NA, Nikon), a 100× Oil objective (1.45 NA, Nikon), a Perfect Focus Unit (Nikon), a Motorized XY stage (Prior Scientific), a fully enclosed and environmentally controlled cage incubator (Okolab), a motorized TIRF Illuminator Unit (Nikon), OBIS 488, 561, and 647 nm lasers (Coherent), the W-VIEW GEMINI Image splitting optics (Hamamatsu) and an EMCCD camera (iXon Life 888, Andor Technology). The 1.5× tube lens equipped on the Nikon TiE microscope was applied for TIRF imaging experiments (final pixel size corresponding to 86.7 nm of the image). The microscope was also equipped with a CSU-X1 spinning disk confocal unit (Yokogawa) and an EMCCD camera (iXon Ultra 897, Andor Technology) on the left side port. Imaging sequences were acquired using Micro-Manager 2.0^71^. The genome-edited SK-BR-3 cells expressing EGFR-mEGFP or HER2-mEGFP were imaged by spinning disk confocal microscopy (3 imaging planes spaced at 0.75 μm, exposure time 100 ms) with a 100× oil objective (1.45 NA, Nikon) every 2 min for 32 min. The genome-edited SK-BR-3 cells expressing EGFR-mEGFP or HER2-mEGFP were also stably expressed with clathrin-mScarlet-I, and then imaged at the bottom surface by TIRF microscopy every 10 s for 12 min with an exposure time 100 ms. EGF was added to the cells at 2 min during imaging by a Syringe Pump (Harvard Apparatus).

### Knockdown of HER2 expression by siRNA

Knockdown of HER2 expression in SK-BR-3 cells genome-edited for EGFR-mEGFP or HER2-mEGFP and stably expressing clathrin-mScarlet-I were achieved by two sequential transfections of siRNA using Lipofectamine RNAiMAX (Invitrogen). The cells plated overnight were transfected with siRNA targeting HER2 (5’-AAATTCCAGTGGCCATCAA-3’) or control siRNA (a mixture of three non-targeting sequences) and then transfected again two days afterward. The cells were transfected with the siRNA-resistant plasmids HER2-Halo or HER2-GS-Halo one day after the second transfection. The siRNA-resistant plasmids bearing mutations (5′-AGATCCCCGTCGCTATCAA-3′) were generated using PCR. Knockdown efficiency was confirmed by western blot analysis. The cells were used for imaging analysis the next day by TIRF microscopy. The cells were imaged every 10 s for 73 frames with an exposure time of 100 ms. EGF was added to the cells at 2 min during imaging by a Syringe Pump (Harvard Apparatus). The two-color time-lapse images were subjected to background subtraction (Rolling ball radius 25 pixels) and cell mask creation by Fiji. The numbers of fluorescent spots of EGFR-mEGFP that appeared at the plasma membrane in each frame during EGF stimulation were detected on the mEGFP channel by batch processing in TrackMate 7 (Fiji)^76,77^. To track the change of EGFR-mEGFP fluorescence at clathrin-coated structures during EGF stimulation, the clathrin-coated structures on the mScarlet-I channel in each frame were detected by batch processing in TrackMate 7, and then the fluorescent intensity of each detected object in the mEGFP channel was measured and plotted.

### Measurement of EGF uptake by spinning disk confocal microscopy

SK-BR-3 cells treated with control siRNA or siRNA targeting HER2 were incubated with Alexa Fluor 488 conjugated EGF (Thermo Fisher; 10 ng/mL) for 10 min or 15 min at 37 °C. The surface-bound EGF was removed by ice-chilled PBS and acid wash buffer (0.1 M glycine, 150 mM NaCl, 1 mM MgCl_2_, and 0.125 mM CaCl_2_, pH 2.5). Then the cells were fixed using 4% paraformaldehyde in PBS for 30 min at room temperature, washed with PBS, and then imaged by spinning-disk confocal microscopy (*z* stack spaced at 0.35 μm). The first four imaging planes near the bottom surface were used to create the *z*-stack projection. The projected images were then subjected to background subtraction, cell mask creation, and then mean intensity measurement of each cell by Fiji.

## Supplementary information

Supplementary Information

Supplementary Video S1

Supplementary Video S2

Supplementary Video S3

Supplementary Video S4

## Data Availability

The cryo-EM density maps of EGF- and EREG-bound EGFR/HER2 heterodimers, and EGF-bound EGFR homodimer have been deposited in the Electron Microscopy Data Bank under the accession codes EMD-34744, EMD-34745, and EMD-34746, respectively. Their atomic coordinates have been deposited in the Protein Data Bank under accession codes 8HGO, 8HGP, and 8HGS, respectively.
