# Supplementary material for: Structure and dynamics of the EGFR/HER2 heterodimer
Source: Cell Discov. 2023 Feb 13;9:18. doi: 10.1038/s41421-023-00523-5 (PMC9925823; doi:10.1038/s41421-023-00523-5)
Supplement: Supplementary file 1 — Supplementary Information [file 41421_2023_523_MOESM1_ESM.pdf]

Supplementary Information for

Structure and dynamics of the EGFR/HER2 heterodimer

Authors:

Xue Bai[1#], Pengyu Sun[2,3#], Xinghao Wang[2,3#], Changkun Long[1#], Shuyun Liao[4#], Song Dang[2], Shangshang Zhuang[2,3], Yongtao Du[2,3], Xinyi Zhang[2,3], Nan Li[2], Kangmin He[2,3*], and Zhe Zhang[1,4*]

Affiliations:

[1]State Key Laboratory of Membrane Biology, School of Life Sciences, Peking University, Beijing 100871, China

[2]State Key Laboratory of Molecular Developmental Biology, Institute of Genetics and Developmental Biology, Chinese Academy of Sciences, Beijing 100101, China

[3]University of Chinese Academy of Sciences, Beijing 100049, China

[4]Center for Life Sciences, Academy for Advanced Interdisciplinary Studies, Peking University, Beijing 100871, China

[#]These authors contributed equally

[*]Correspondence should be addressed to zzhang01@pku.edu.cn (Z.Z.) or kmhe@genetics.ac.cn (K.H.).

This file includes:

Supplementary Figures S1 to S8

Supplementary Table S1

Legends for Supplementary Videos S1 to S4

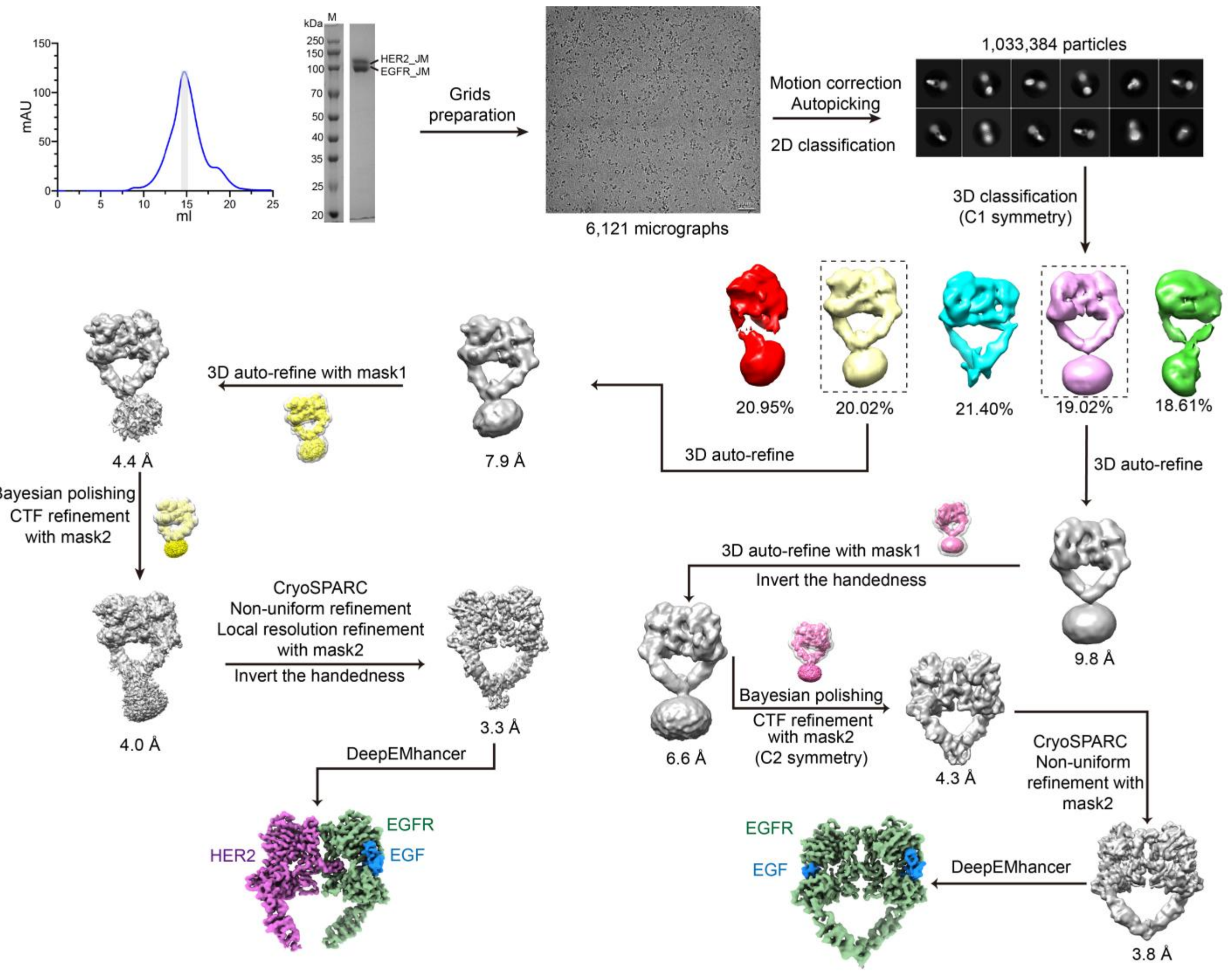

Supplementary Fig. S1 Cryo-EM analyses of the EGFR-EGF/HER2 dataset.

Workflow of the sample preparation and data processing of the EGFR-EGF/HER2 dataset.

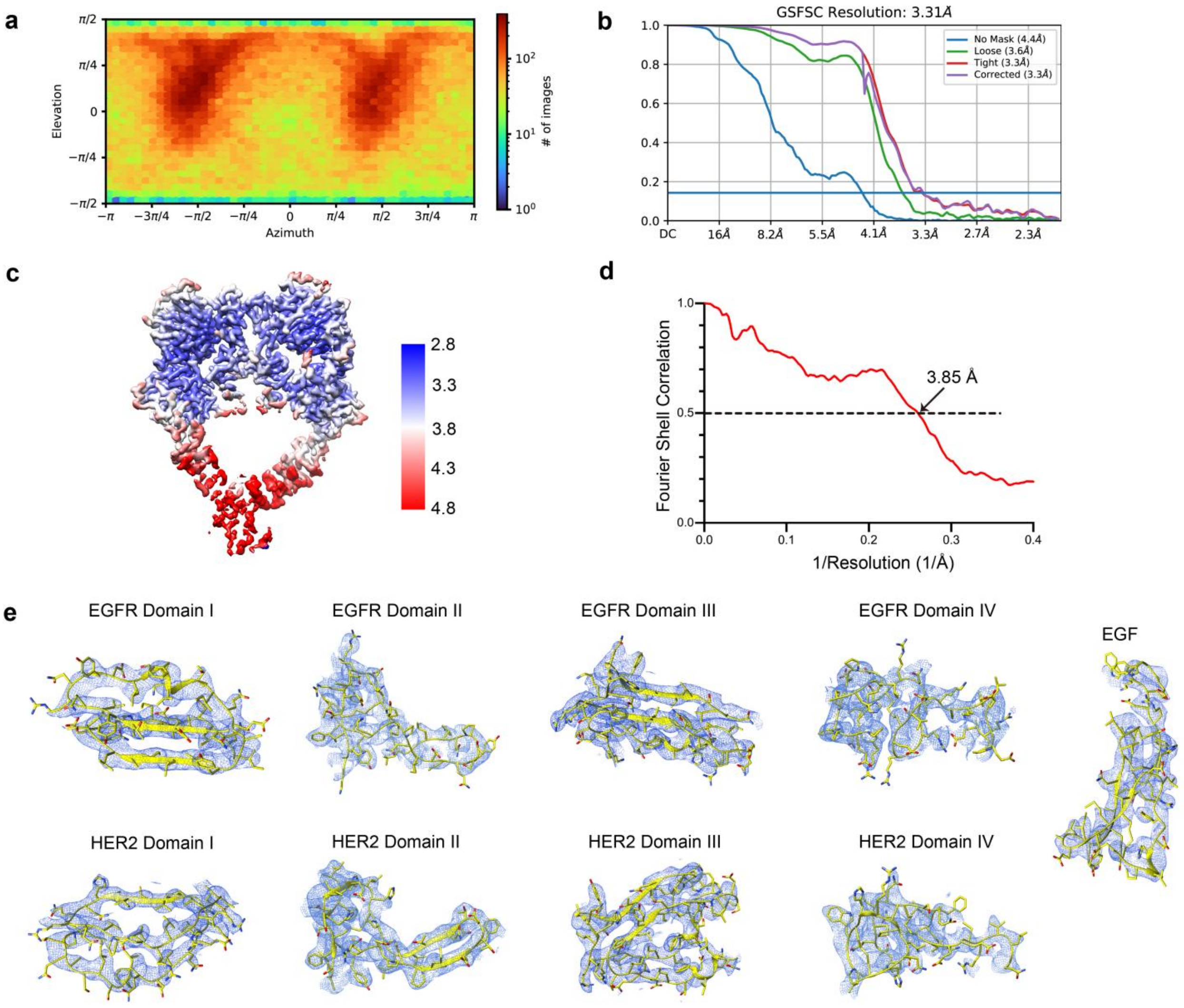

Supplementary Fig. S2 Assessments of the EGFR-EGF/HER2 structure.

a Angular distribution of the particles used in the final reconstruction.

b Fourier shell correlation (FSC) curves between two half maps.

c Local resolution of the cryo-EM map.

d FSC curve calculated between cryo-EM map and structural model.

e Close-up view of the cryo-EM densities and fitted atomic models for representative regions of the structure.

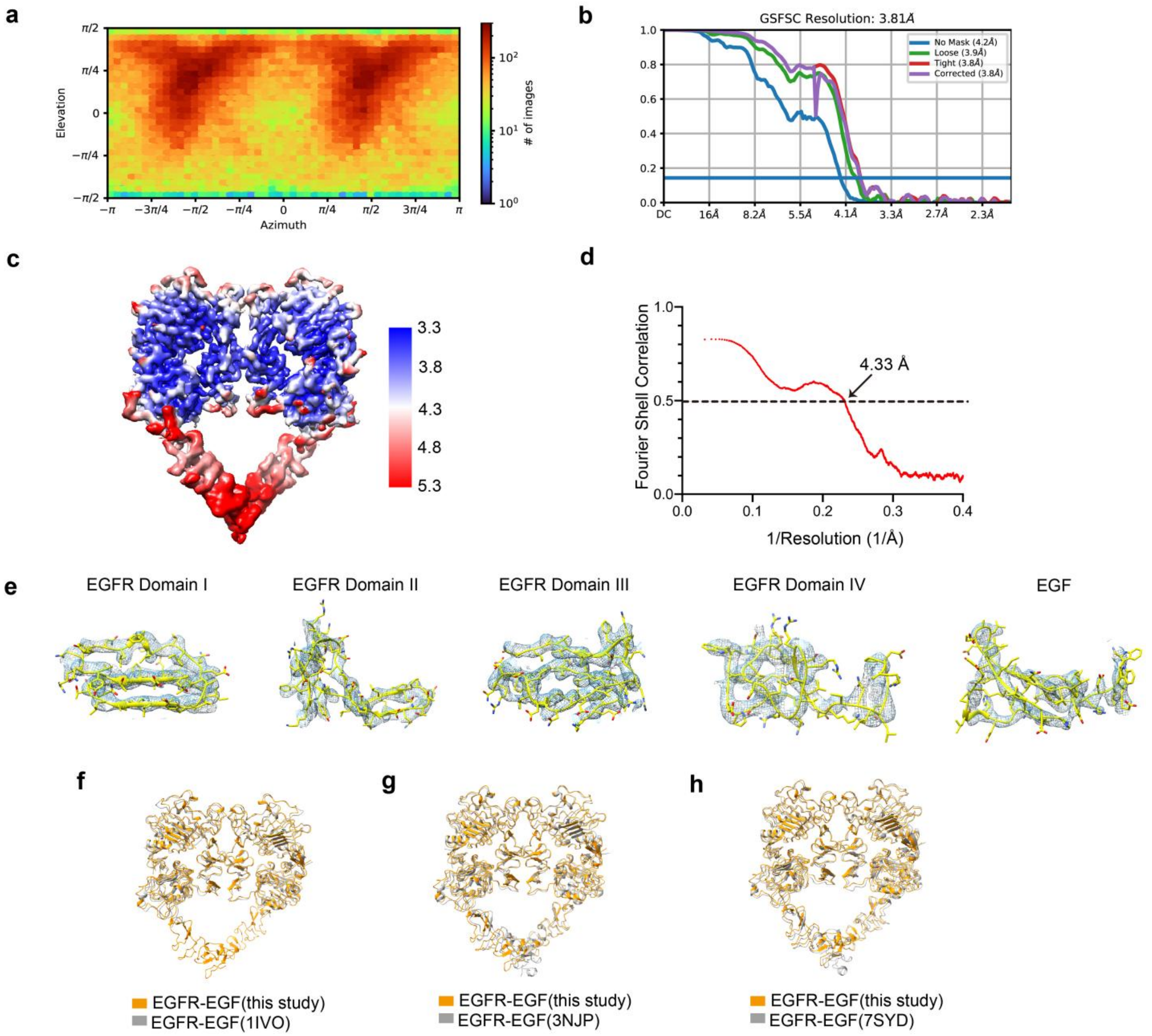

Supplementary Fig. S3 Assessments of the EGFR-EGF homodimer structure.

a Angular distribution of the particles used in the final reconstruction.

b Fourier shell correlation (FSC) curves between two half maps.

c Local resolution of the cryo-EM map.

d FSC curve calculated between cryo-EM map and structural model.

e Close-up view of the cryo-EM densities and fitted atomic models for representative regions of the structure.

f-h Superposition of our EGFR-EGF homodimer structure (golden) with the currently reported three structures (grey). The RMSDs between our structure and the others are 1.45 Å (PDB code 1IVO), 1.34 Å (PDB code 3NJP), and 1.44 Å (PDB code 7SYD), respectively.

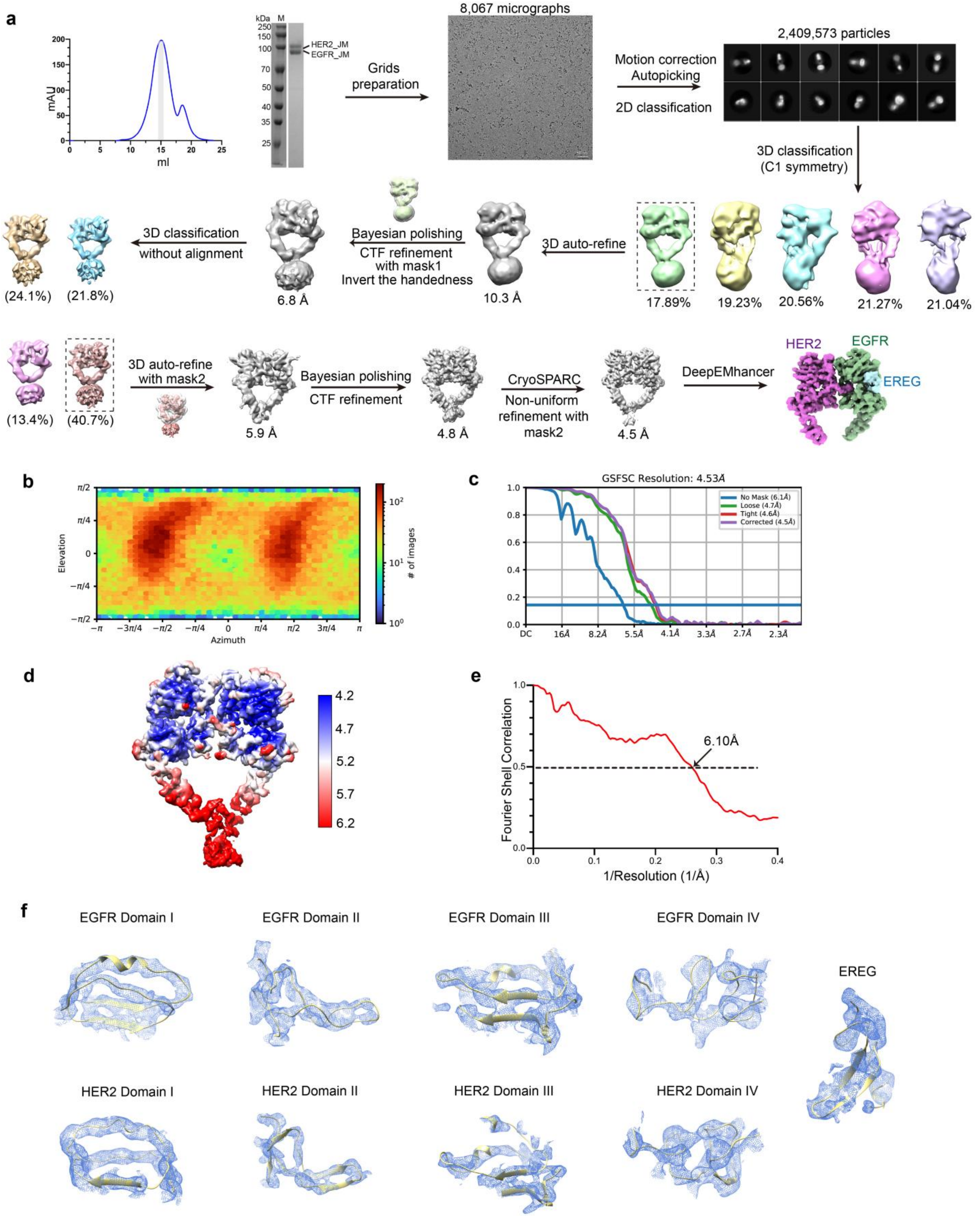

Supplementary Fig. S4 Cryo-EM data processing of the EGFR-EREG/HER2 dataset.

a Workflow of the data processing.

b Angular distribution of the particles used in the final reconstruction.

c Fourier shell correlation (FSC) curves between two half maps.

d Local resolution of the cryo-EM map.

e FSC curve calculated between cryo-EM map and structural model.

f Close-up view of the cryo-EM densities and fitted atomic models for representative regions of the structure.

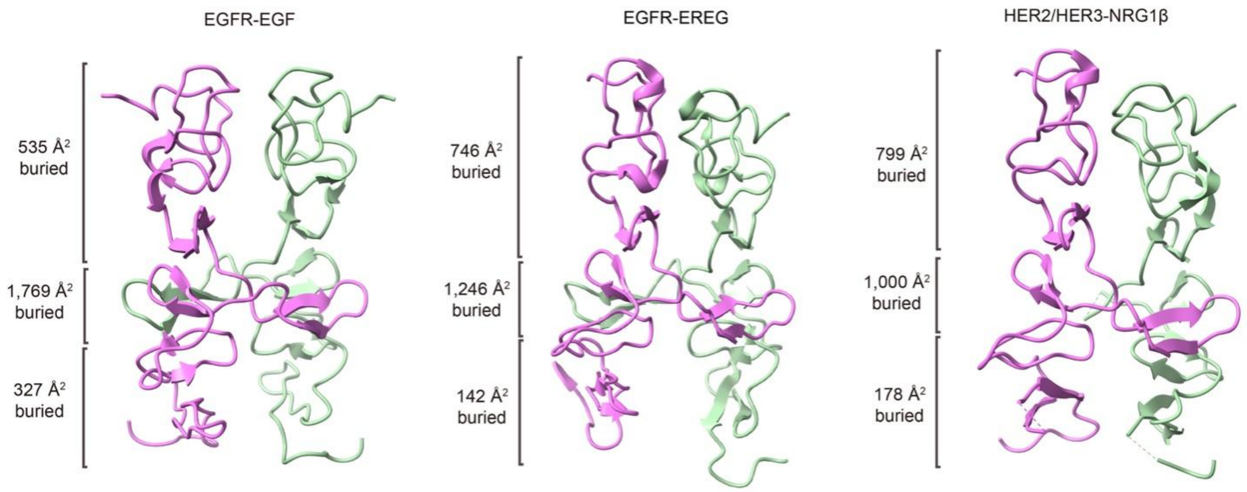

Supplementary Fig. S5 Interaction details of Domain II in different human HER dimers.

From left to right: EGFR-EGF (PDB code 1IVO), EGFR-EREG (PDB code 5WB7), and HER2/HER3-NRG-1β (PDB code 7MN5). The buried surface areas of the dimerization arms, and the N- and C-terminal regions are calculated and indicated separately.

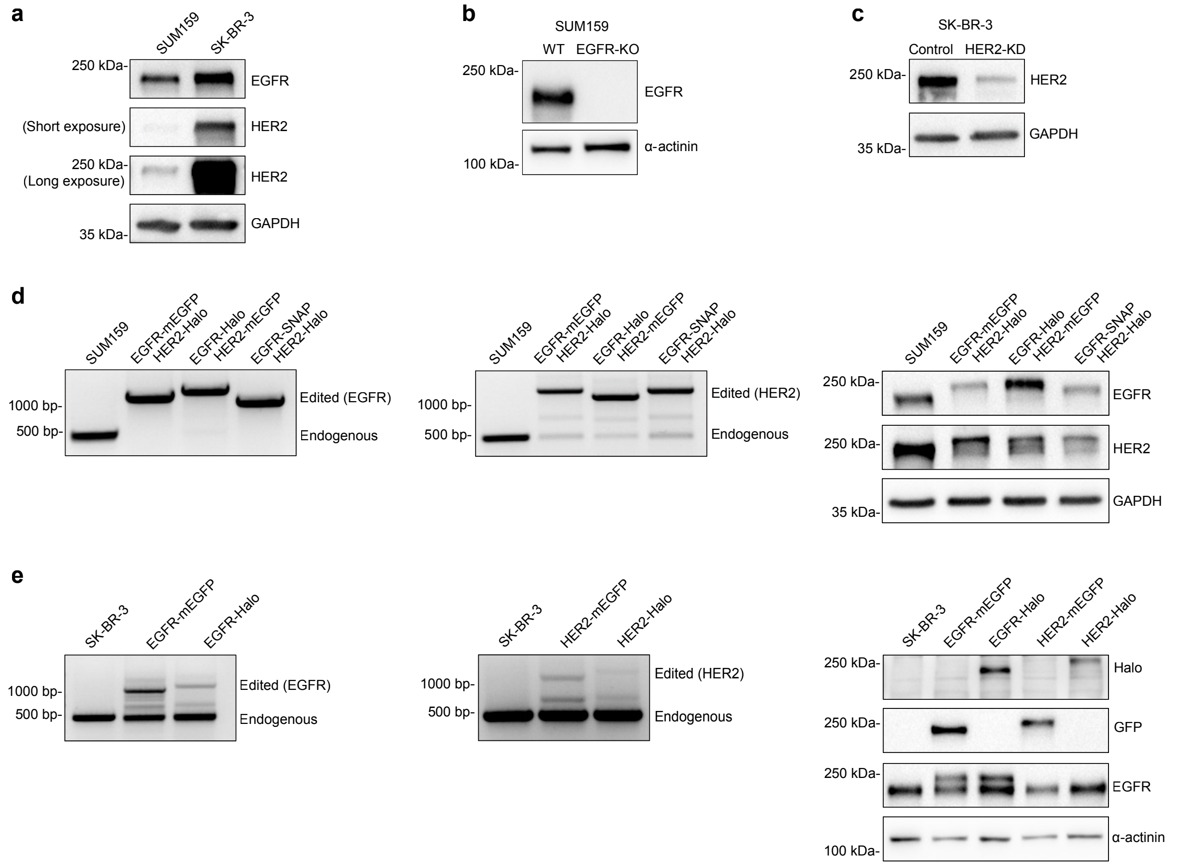

Supplementary Fig. S6 Genome-editing of SUM159 and SK-BR-3 cells.

a Western blot analysis of the relative expression levels of EGFR and HER2 in SUM159 and SK-BR-3 cells.

b Loss of EGFR expression in a clonal SUM159 cell line knocked out of EGFR was confirmed by Western blot.

c Western blot analysis of SK-BR-3 cells treated with control siRNA or siRNA specific for HER2.

d Genomic PCR analysis showing the integration of mEGFP, HaloTag, or SNAP-tag to the EGFR or HER2 genomic locus of SUM159 cells to generate three genome-edited cell lines (pool) (left and middle panels). Western blot analysis of the cell lines was shown on the right.

e Genomic PCR analysis showing the integration of mEGFP or HaloTag to the EGFR or HER2 genomic locus of SK-BR-3 cells to generate four genome-edited cell lines (pool) (left and middle panels). Western blot analysis of the cell lines was shown on the right.

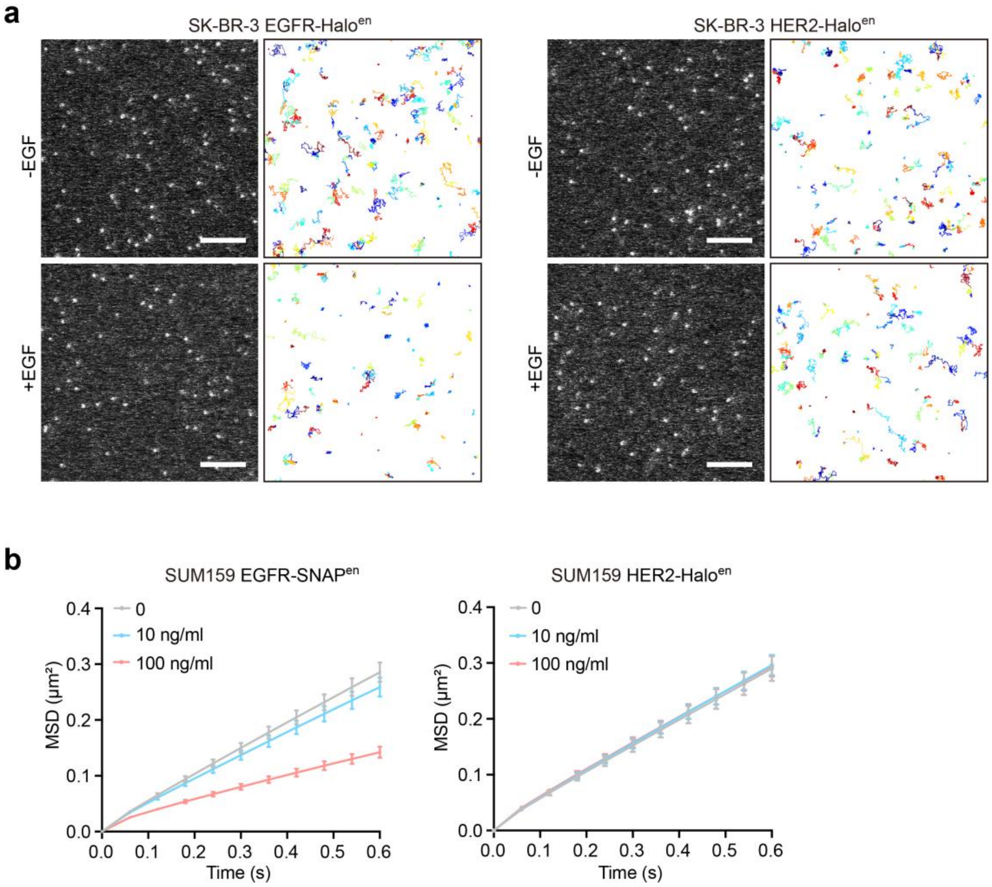

Supplementary Fig. S7 Diffusion dynamics of EGFR and HER2.

a Live-cell single-molecule imaging and tracking of endogenous EGFR-Halo and HER2-Halo molecules at the plasma membrane of SK-BR-3 cells genome-edited for EGFR-Halo or HER2-Halo (labeled by JFX_{650}-HaloTag ligand). Shown are representative single frames and tracking traces of time-lapse series acquired in the cells treated without or with EGF (100 ng/mL) by TIRF microscopy. Scale bars, 5 μm.

b SUM159 cells genome-edited for both EGFR-SNAP and HER2-Halo were labeled by JFX_{650}-SNAP-tag ligand and JFX_{549}-HaloTag ligand, treated with 0, 10, or 100 ng/mL EGF, and then imaged by TIRF microscopy. Shown are MSD-Δt plots of EGFR-SNAP tracks (left pane) or HER2-Halo tracks (right pane) (n = 57, 62, and 63 cells). Error bars show mean ± 95% confidence interval.

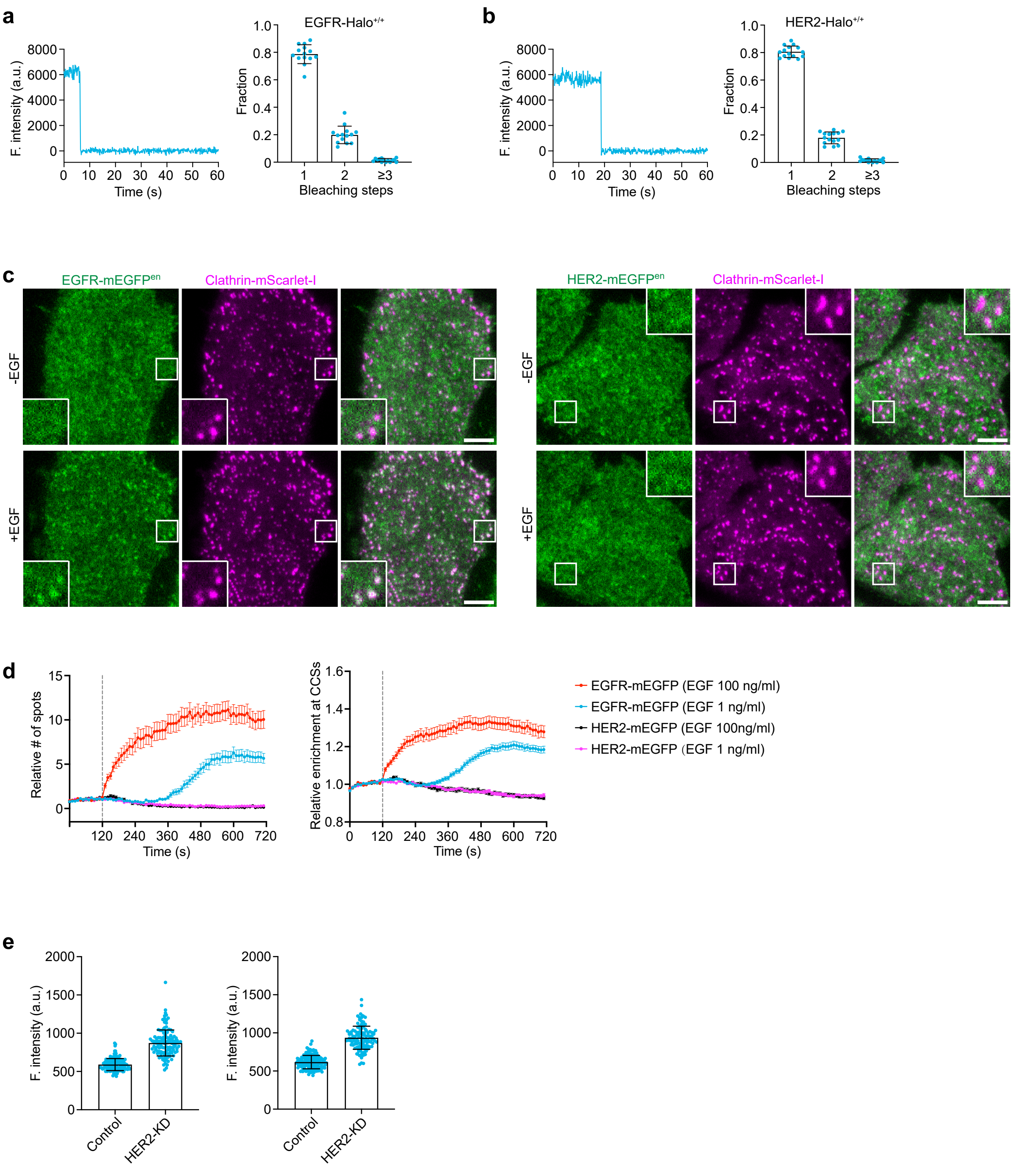

Supplementary Fig. S8 Comparison of EGFR and HER2 endocytosis.

a, b SUM159 cells genome-edited to express EGFR-Halo$^{+/+}$ or HER2-Halo$^{+/+}$ were labeled by JFX$_{650}$-HaloTag ligand, fixed, and then imaged by continuous TIRF illumination with high laser power. Shown are the fluorescence intensity trace of a single EGFR-Halo/HER2-Halo spot (single-

step bleaching with background subtracted) and the fraction of bleaching steps of single EGFR-Halo (**a**) and HER2-Halo (**b**) spots (n = 14 and 15 cells, mean ± SD).

c SK-BR-3 cells genome-edited for EGFR-mEGFP (left panel) or HER2-mEGFP (right panel) were stably expressed with clathrin-mScarlet-I and imaged at the bottom surfaces by TIRF microscopy. Shown are the single frames before and 3 min after EGF treatment (1 ng/mL) during the continuous time-lapse imaging. Boxed regions are enlarged and shown. Scale bars, 5 μm.

d SK-BR-3 cells genome-edited for EGFR-mEGFP or HER2-mEGFP were stably expressed with clathrin-mScarlet-I and imaged at the bottom surfaces by TIRF microscopy. EGF (1 ng/mL or 100 ng/mL) was added at 120 s of the time-lapse imaging. The relative numbers of fluorescence spots of EGFR-mEGFP or HER2-mEGFP that appeared at the plasma membrane (left panel, n = 30, 37, 37, and 41 cells, mean ± SEM) and the relative enrichment of EGFR-mEGFP or HER2-mEGFP fluorescence in clathrin-coated structures (CCSs, right panel, n = 30, 37, 23, and 37 cells, mean ± SEM) during EGF stimulation are shown. The continuous imaging caused slight photobleaching of HER2-mEGFP at the plasma membrane.

e SK-BR-3 cells were treated with control siRNA or siRNA targeting HER2 (HER2-KD), and then incubated with Alexa Fluor 488 conjugated EGF for 10 min (left panel) or 15 min (right panel), fixed and then imaged by spinning-disk confocal microscopy (Z stack spaced at 0.35 μm). The fluorescence intensity of Alexa Fluor 488 conjugated EGF in the control and HER2-KD cells were measured and shown (10 min: 137 control and 127 KD cells; 15 min: 150 control and 118 KD cells; mean ± SD).

Supplementary Table S1 Cryo-EM data collection, refinement, and validation statistics.

	EGFR-EGF/HER2 (EMD-34744) (PDB 8HGO)	EGFR-EREG/HER2 (EMD-34745) (PDB 8HGP)	EGFR-EGF dimer (EMD-34746) (PDB 8HGS)
**Data collection and processing**			
Magnification	81,000	81,000	81,000
Voltage (kV)	300	300	300
Electron exposure ($e^{-}/Å^2$)	60.8	60.8	60.8
Defocus range (μm)	1.2-1.5	1.2-1.5	1.2-1.5
Pixel size (Å)	1.07	1.07	1.07
Symmetry imposed	C1	C1	C2
Initial particle images (no.)	1,990,190	2,409,573	1,990,190
Final particle images (no.)	207,092	273,388	196,557
Map resolution (Å)	3.31	4.53	3.81
FSC threshold	0.143	0.143	0.143
Map resolution range (Å)	2.3-15	2.9-15	3.2-15
**Refinement**			
Initial model used (PDB code)	7MN5/1IVO	7MN5/5WB7	7SYD
Model resolution (Å)	3.85	6.10	4.33
FSC threshold	0.5	0.5	0.5
Model resolution range (Å)	-	-	-
Map sharpening *B* factor ($Å^2$)	-	-	-
Model composition			
Non-hydrogen atoms	9,227	9,169	10,080
Protein residues	1,178	1,172	1,280
Ligands	-	-	-
B factors ($Å^2$)			
Protein	78.79	185.39	111.68
Ligand	-	-	-
R.m.s. deviations			
Bond lengths (Å)	0.003	0.003	0.003
Bond angles (°)	0.702	0.670	0.732
Validation			
MolProbity score	1.72	1.69	1.61
Clashscore	4.51	3.98	3.29
Poor rotamers (%)	0.00	0.20	0.00
Ramachandran plot			
Favored (%)	91.80	91.49	91.90
Allowed (%)	8.03	8.51	8.10
Disallowed (%)	0.17	0.00	0.00

Supplementary Video S1 Live-cell single-molecule imaging and tracking of endogenous EGFR-Halo at the plasma membrane of SUM159 cells treated without or with EGF (100 ng/mL) and imaged by TIRF microscopy.

Supplementary Video S2 Live-cell single-molecule imaging and tracking of endogenous HER2-Halo at the plasma membrane of SUM159 cells treated without or with EGF (100 ng/mL) and imaged by TIRF microscopy.

Supplementary Video S3 Live-cell single-molecule imaging and tracking of endogenous EGFR-Halo at the plasma membrane of SK-BR-3 cells treated without or with EGF (100 ng/mL) and imaged by TIRF microscopy.

Supplementary Video S4 Live-cell single-molecule imaging and tracking of endogenous HER2-Halo at the plasma membrane of SK-BR-3 cells treated without or with EGF (100 ng/mL) and imaged by TIRF microscopy.
